# Effects of icosapent ethyl according to baseline residual risk in patients with atherosclerotic cardiovascular disease: results from REDUCE-IT

**DOI:** 10.1093/ehjcvp/pvae030

**Published:** 2024-04-27

**Authors:** Pascal M Burger, Deepak L Bhatt, Jannick A N Dorresteijn, Stefan Koudstaal, Arend Mosterd, Fabrice M A C Martens, Philippe Gabriel Steg, Frank L J Visseren, for the REDUCE-IT Investigators, Joanne Ladner, Lily Kakish, Ashley Kakish, Amy L Little, Jaime Gerber, Nancy J Hinchion, Janet Guarino, Denise Raychok, Susan Budzinski, Kathleen Kelley-Garvin, April Beckord, Jessica Schlinder, Arthur Schwartzbard, Stanley Cobos, Deborah Freeman, David Abisalih, Dervilla McCann, Kylie Guy, Jennifer Chase, Stacey Samuelson, Madeline Cassidy, Marissa Tardif, Jaime Smith, Brenna Sprout, Nanette Riedeman, Julie Goza, Lori Johnson, Chad Kraske, Sheila Hastings, Chris Dutka, Stephanie Smith, Toni McCabe, Kathleen Maloney, Paul Alfieri, Vinay Hosemane, Chanhsamone Syravanh, Cindy Pau, April Limcoiloc, Tabitha Carreira, Taryn S Kurosawa, Taryn S Kurosawa, Razmig Krumian, Krista Preston, Ashraf Nashed, Daria Schneidman-Fernandez, Jack Patterson, John Tsakonas, Jennifer Esaki, Lynn Sprafka, Porous Patel, Brian Mitchell, Erin M Ross, Donna Miller, Akash Prashad, Kristina M Feyler, Natasha Juarbe, Sandra Herrera, Sarah M Keiran, Becky Whitehead, Whitney Asher, Coury Hobbs, Abbey Elie, Jean Brooks, Amanda L Zaleski, Brenda Foxen, Barb Lapke, Philippa Wright, Bristol Pavol, Gwen Carangi, Marla Turner, Howard S Ellison, Katharine W Sanders, Rikita S Delamar, Virginia L Wilson, Sarah M Harvel, Alison M Cartledge, Kaitlyn R Bailey, Kathleen Mahon, Timothy Schuchard, Jen Humbert, Mark C Hanson, Michael P Cecil, James S Abraham, Lorie Benedict, Claudia Slayton, Curtis S Burnett, Rachel W Ono-Lim, Sharon Budzinski, Shubi A Khan, Sharon Goss, Terry Techmanski, Farida Valliani, Rimla Joseph, Edith Flores, Laurn Contreras, Ana Aguillon, Carrie-Ann Silvia, Maria Martin, Edmund K Kerut, Leslie W Levenson, Louis B Glade, Brian J Cospolich, Maureen W Stein, Stephen P LaGuardia, Thelma L Sonza, Tracy M Fife, Melissa Forschler, Jasmyne Watts, Judy Fritsch, Emese Futchko, Sarah Utech, Scott B Baker, Miguel F Roura, David R Sutton, Scott A Segel, James S Magee, Cathy Jackson, Rebecca F Goldfaden, Liudmila Quas, Elizabeth C Ortiz, Michael Simpson, Robert Foster, C Brian, James Trimm, Michael Bailey, Brian Snoddy, Van Reeder, Rachel Wilkinson, Harold Settle, Cynthia Massey, Angela Maiola, Michele Hall, Shelly Hall, Wanda Hall, Mark Xenakis, Janet Barrett, Giovanni Campanile, David Anthou, Susan F Neill, Steven Karas, Enrique Polanco, Norberto Schechtman, Grace Tischner, Kay Warren, Cynthia St Cyr, Menna Kuczinski, Latrina Alexander, Maricruz Ibarra, Barry S Horowitz, Jaime Steinsapir, Jeanette Mangual-Coughlin, Brittany Mooney, Precilia Vasquez, Kathleen Rodkey, Alexandria Biberstein, Christine Ignacio, Irina Robinson, Marcia Hibberd, Lisa B Hoffman, Daniel J Murak, Raghupathy Varavenkataraman, Theresa M Ohlson Elliott, Linda A Cunningham, Heather L Palmerton, Sheri Poole, Jeannine Moore, Helene Wallace, Ted Chandler, Robert Riley, Farah Dawood, Amir Azeem, Michael Cammarata, Ashleigh Owen, Shivani Aggarwal, Waqas Qureshi, Mohamed Almahmoud, Abdullahi Oseni, Adam Leigh, Erin Barnes, Adam Pflum, Amer Aladin, Karen Blinson, Vickie Wayne, Lynda Doomy, Michele Wall, Valerie Bitterman, Cindi Young, Rachel Grice, Lioubov Poliakova, Jorge Davalos, David Rosenbaum, Mark Boulware, Heather Mazzola, J Russell Strader, Russell Linsky, David Schwartz, Elizabeth Graf, Alicia Gneiting, Melissa Palmblad, Ashley Donlin, Emily Ensminger, Hillary Garcia, Dawn Robinson, Carolyn Tran, Jeffrey Jacqmein, Darlene Bartilucci, Michael Koren, Barbara Maluchnik, Melissa Parks, Jennifer Miller, Cynthia DeFosse, Albert B Knouse, Amy Delancey, Stephanie Chin, Thomas Stephens, Mag Sohal, Juana Ingram, Swarooparani Kumar, Heather Foley, Nina Smith, Vera McKinney, Linda Schwarz, Judith Moore, H V Anderson, Stefano SdringolaMaranga, Ali Denktas, Elizabeth Turrentine, Rhonda Patterson, John Marshall, Terri Tolar, Donna Patrick, Pamela Schwartzkopf, Anthony M Fletcher, Frances R Harris, Sherry Clements, Tiffany Brown, William Smith, Stacey J Baehl, Robin Fluty, Daniel VanHamersveld, Dennis Breen, Nancy Bender, Beverly Stafford, Tamika Washington, Margaret N Pike, Mark A Stich, Evyan Jawad, Amin Nadeem, Jill Nyland, Rhonda Hamer, Kendra Calhoun, Charlotte Mall, Samuel Cadogan, Kati Raynes, Richard Katz, Lorraine Marshall, Rashida Abbas, Jay L Dinerman, John T Hartley, Beth Lamb, Lisa Eskridge, Donna Raymond, Kristy Clemmer, Denise M Fine, Paula Beardsley, Janet Werner, Bette Mahan, Courtney VanTol, Robert Herman, Christine Raiser-Vignola, Felicia McShan, Stefanie A Neill, David R Blick, Michael J Liston, Denetta K Nelson, Sandra K Dorrell, Patricia Wyman, Ambereen Quraishi, Fernando Ferro, Frank Morris, Vicki J Coombs, Autumn M Mains, Austin A Campbell, Jeanne Phelps, Cheryl A Geary, Ellen G Sheridan, Jean M Downing, Arie Swatkowski, Tish Redden, Brian Dragutsky, Susan Thomas, Candace Mitchell, Diana Barker, Elanie Turcotte, Deborah Segerson, Jill Guy, Karena De La Mora, Jennifer Hong, Dennis Do, Rose Norris, Faisal Khan, Hector Montero, Stacy Kelly-White, Alan Cleland, Rosalyn Alcalde-Crawford, Melissa Morgan, Brijmohan Sarabu, Megan Minor, Shweta Kamat, Stephanie M Estes, Nancee Harless, Alicia Disney, Jodi L Pagano, Chad M Alford, Noel W Bedwell, Warren D Hardy, Kevin DeAndrade, Jessica G Elmore, Eric G Auerbach, Anthony W Haney, Miriam H Brooks, Jose Torres, Lois Roper, Terry Backer, Katie Backer, Lois Roper, John G Evans, James S Magee, Ricardo A Silva, Lorraine H Dajani, Scott A Segel, Veronica Yousif, Tammy Ross, Rebecca F Goldfaden, Sion K Roy, Ronald Oudiz, Sajad Hamal, Ferdinand Flores, Amor Leahy, Debra Ayer, Swapna George, Chrisi Carine Stewart, Elvira Orellana, Cristina Boccalandro, Mary Rangel, Suzanne Hennings, Carl Vanselow, Teri Victor, Darlene Birdwell, Paul Haas, Anthony Sandoval, Gina Ciavarella, Caroline Saglam, Amy Bird, Keith Beck, Brian Poliquin, David Dominguez, Brittany Tenorio, Harvonya Perkins, Esther San Roman, Paris Bransford, Christy Lowrance, Marcy Broussard, Mary Ellis, Bobbi Skiles, Jessica Hamilton, Kathryn Hall, Diego Olvera, Kathleen Maloney, Julee A Hartwell, Nevien Sorial, Kishlay Anand, Mary Rickman, Kevin Berman, Nirav Mehta, Annie Laborin, Caroline Saglam, Rodger Rothenberger, Sarah Beauvilliers, Kathy Morrell, Michael P Schachter, Cindy L Perkins, Elizabeth A Gordon, Jennifer Lauer, Kim Bichsel, Kelly Oliver, Leslie J Mellor, Candice Demattia, Jennifer Schomburg, Yenniffer Moreno, Eduardo Mansur-Garza, Lena Rippstein, Lorie Chacon, Andrea Pena, Michelle King, Susan Richardson, Annette Jessop, Nicole Tucker, Whitney Royer, Gilbert Templeton, Ann Moell, Christine Weller, Melissa J Botts, Gretel Hollon, Elsa Homberg-Pinassi, Paula Forest, Aref Bin Abhulhak, Devona Chun-Furlong, Deborah Harrington, Emily Harlynn, Marjorie Schmitt, Constance Shelsky, Patricia Feldick, Mary Cherrico, Courtney Jagle, Nicholas Warnecke, Debra Myer, Deanna J Ruder, Albina Underwood, Alan Rauba, George Carr, Barbara Oberhaus, Jessica Vanderfeltz, Mary Jo Stucky-Heil, Dale R Gibson, Vonnie Fuentes, Kimberly L Talbot, William C Simon, Katlyn J Grimes, Christina R Wheeler, Cassaundra Shultz, Rhonda A Metcalf, Jennifer L Hill, Michelle R Oliver, Basharat Ahmad, Fouzal Azeem, Abdul Rahim, George H Freeman, Dawn Bloch, Heather Freeman, Jamie Brown, Sarah Rosbach, Pamela Melander, Nick Taralson, Alex Liu, Katlyn Harms, Mahfouz Michale, Jose Lopez, Maria Revoredo, Shari Edevane, Sarah Shawley, Timothy L Jackson, Michael J Oliver, Dina DeSalle, Patricia J Matlock, Ionna M Beraun, Heather Hendrix, Garrett Bromley, Ashley Niemerski, Gabby Teran, Sonia Guerrero, Murtaza Marvi, Zehra Palanpurwala, Andrea Torres, Patty Gloyd, Michelle Conger, Andrea Pena, Aziz Laurent, Olia Nayor, Catalina S Villanueva, Munira Khambati, Tabetha J Mumford, Melanie J Castillo, Taddese Desta, Jerome Robinson, La Shawn Woods, Anita H Bahri, Nancy Herrera, Cecilia Casaclang, Jeffrey R Unger, Geraldine Martinez, Mia K Moon, Stephen M Mohaupt, Larry Sandoval, Louisito Valenzuela, Victora Ramirez, Nelly Mata, Veronica Avila, Marisol Patino, Cynthia Montano-Pereira, Omar Barnett, William M Webster, Lorraine M Christensen, Leighna Bofman, Melanie Livingston, Stacey Adams, Joseph Hobbs, Stacey Adams, Leesa Koskela, Mia Katz, Samuel Mujica-Trenche, Franklin Cala, Noreen T Rana, Jennifer Scarlett, Milagros Cala Anaya, Marsha R Jones, Kelly D Hollis, Debbie Roth, Kristin Eads, Tina Watts, Judy Perkins, Alice Arnold, Daniel C Ginsberg, Denise Quinn, Nicole Cureton, David B Fittingoff, Mohammed I Iqbal, Stephen R White, Edith Sisneros, Michelle Ducca, David Streja, Danny Campos, Jennifer L Boak, Farzeen Amir, Felice Anderson, James J Kmetzo, Mary O Bongarzone, Dawn Scott, Mary Grace De Leon, Cynthia Buda, William Graettinger, Michelle Alex, William Graettinger, Erika Hess, James Govoni, Melissa Bartel, Travis L Monchamp, Julie S Roach, Sara Gibson, Amy M Allfrey, Kristen Timpy, Kathy Bott, Karin A Soucy, Jean Willis, Cecilia A Valerio, Anusha Chunduri, Rebecca Coker, Nicole Vidrine, Ellen A Thompson, Mark A Studeny, Melissa K Marcum, Tammy S Monway, Douglas L Kosmicki, Melissa J Kelley, Corey M Godfrey, Susan L Krenk, Randy R Holcomb, Deb K Baehr, Mary K Trauernicht, David Rowland Lowry, Betty Bondy Herts, Jeanne E Phelps, Jean-Marie Downing, Carol Gamer Dignon, Elisabeth S Cockrill, Pravinchandra G Chapla, Diane Fera, Margaret Chang, Patricia Fredette, Tamie Ashby, Renee Bergin, Zebediah A Stearns, David B Ware, Rachael M Boudreaux, Francisco G Miranda, Joanna Rodriguez, Robert McKenzie, Amanda Huber, Rebecca Sommers, Heather Rowe, Stacy McLallen, Michale Haynes, Ashley Adamson, Janice Henderson, Lori McClure, Beverly A Harris, Laura Ference, Sue Meissner-Dengler, Lisa Treasure, Doreen Nicely, Timothy L Light, Tracey A Osborn, Kimberly J Mai, Pablo Vivas, Jose Rios, Dunia Rodriguez, Roger DeRaad, James Walder, Oscar Bailon, Denice Hockett, Debbie Anderson, Kelli McIntosh, Amber Odegard, Andrew Shepherd, Mary Seifert, Laurence Kelley, Rajendra Shetty, Michael Castine, David Brill, Gregory Fisher, Nicole Richmond, Kathleen Gray, Patricia Miller, Charlene Coneys, Yarixa Chanza, Monica Sumoza, Victoria M Caudill, Kelly D Harris, Courtney A Manion, Melody J Lineberger-Moore, Julie J Wolfe, Barbara J Rosen, Patricia DiVito, Janet L Moffat, Christina Michaelis, Prashant Koshy, Diana Perea, Ghaith Al Yacoub, Stephanie Sadeghi, Thomas D LeGalley, Rudolph F Evonich, William J Jean, Gary M Friesen, John M Pap, David A Pesola, Mark D Cowan, Kristofer M Dosh, Dianna Larson, Adele M Price, Jodi A Nease, Jane E Anderson, Lori A Piggott, Robert Iwaoka, Kevin Sharkey, Edward McMillan, Laurie Lowder, Latisha Morgan, Kyle Davis, Tara Caldwell, Erica Breglio, Jasmine Summers, Rachel Poulimas, Muhammad Zahid, Hamid Syed, Maria Escobar, Jacob Levy, Rahma Warsi, Carol Ma, Puxiao Cen, Kimberly A Cawthon, Delores B Barnes, Deanna G Allen, Margaret L Warrington, Carol R Stastny, Robin J Michaels, Mohamad Saleh, John Sorin, Sunny Rathod, Urakay Juett, Steven Spencer, Aziza Keval, Jill McBride, Shane Young, Catherine Baxter, Carol Rasmussen, Shari L Coxe, Luis Campos, Shahin Tavackoli, Diana Beckham, Darlynee Sanchez, Karanjit Basrai, Dorian Helms, Erica Clinton, Kasie Smith, Arnold Ghitis, Henry Cusnir, Mary Klaus Clark, Madhavagopal V Cherukuri, Ameta Scarfaru, Stephen D Nash, Jeannine Moore, Helene Wallace, Loretta C Grimm, Anna Grace, Kylie McElheran, Dino Subasic, Zedrick Buhay, Janet Litvinoff, Tamika Washington, Deepak Shah, Shannon Cervantes, Freda Usher, Farra Yasser, Theodore Trusevich, Ronnie L Garcia, Jamison Wyatt, Rahul Bose, Holllilyn Miska, Traci Spivey, Amy B Wren, Katie E Vance, Lani L Holman, Pam Gibbons, Elaine Eby, Sandra Shepard, Soratree Charoenthongtrakul, Brett Snodgrass, Mohammed Nazem, Shelly Keteenburg, Prathima Murthy, Frederic Prater, Ashley Rumfelt, Christina Eizensmits, Lisa Iannuzzi, Pourus R Patel, Clellia Bergamino, Elizabeth McFeaters, Botros Rizk, Emiljia Pflaum, Danny Kalish, Rex Ambatali, Mona Ameli, Delaina Sanguinetti, Mona Ameli, Rakesh Vaidya, Karen Blinson, Lynda Doomy, Vickie Wayne, Rob J Bos, Alexandra Wils, Tamara Jacobs, Erik A Badings, Lillian A Ebels-Tuinbeek, Mayke L Scholten, Esther BayraktarVerver, Debby Zweers, Manoek Schiks, Carolien Kalkman, Tineke Tiemes, Jeanette Mulderij, W Hermans, K Dabrowska, W Wijnakker, R Vd Loo, J DeGraauw, Giny Reijnierse, Mirjam van der Zeijst, Mariska Scholten, Henk R Hofmeijer, Antoinette van Dijk-van der Zanden, Dineke J van Belle, Jan Van Es, Gera Van Buchem, Wendy Zijda, Harald Verheij, Linnea Oldenhof-Janssen, Martina Bader, Marije Löwik, Sandra Stuij, Pascal Vantrimpont, Krista van Aken, Karen Hamilton, Arno van der Weerdt, Han Blömer, Gabriela van Laerhoven, Raymond Tukkie, Maarten Janssen, Gerard Verdel, Jon Funke Küpper, Bob van Vlies, Caroline Kalkman, Joke Vooges, Marinella Vermaas, Jeanne de Graauw, Riny Van de Loo, Rachel Langenberg, Niek Haenen, Frans Smeets, Arko Scheepmaker, Marcel Grosfeld, Ilvy Van Lieshout, Marleen van den Berg, Marian Wittekoek, Petra Mol, Antionette Stapel, M Sierevogel, N vd Ven, A Berkelmans, Eric Viergever, Hanneke Kramer, Wilma Engelen, Karen V Houwelingen, Thierry X Wildbergh, Arend Mosterd, Coriet Hobé-Rap, Marjan van Doorn, Petra Bunschoten, Michel Freericks, Mireille Emans, Petra Den Boer-Penning, Els Verlek, Christine Freericks, Cornelis de Nooijer, Christina Welten, Ingrid Groenenberg, C van der Horst, E Vonk, Geert Tjeerdsma, Gerard M Jochemsen, Corinne van Daalen, Ingrid Y Danse, Lucy Kuipers, Anke Pieterse, Antonius Oomen, Daan de Waard, Willem Jan Flu, Zusan Kromhout, Petra Van der Bij, Rob Feld, Brigitta Hessels-Linnemeijer, Rob Lardinois, Jan L Posma, Zwanette R Aukema-Wouda, Marjolijn Hendriks-van Woerden, Desiree van Wijk, Driek P Beelen, Ingrid H Hendriks, Jan J Jonker, Stefanie Schipperen, Vicdan Köse, Gloria Rojas, Linda Goedhart, Hanneke van Meurs, Rachel Langenberg, Jacqueline Rijssemus, Jacqueline Hoogendijk, Lindy Swinkels-Diepenmaat, Wouter van Kempen, Marloes de Louw-Jansen, Dominique Bierens-Peters, Willem W van Kempen, Marianne E Wittekoek, Irmaina Agous, Geert Schenk, Willem W van Kempen, Janneke Wittekoek, Kevin Cox, Deborah F Julia, Jan J C Jonker, Roel Janssen, Willem W van Kempen, Marianne E Wittekoek, Melchor Nierman, Hilligje Katerberg, Jan J C Jonker, Irene van der Haar, Willem W Van Kempen, Taco van Mesdag, Janneke Wittekoek, Jan J C Jonker, Leyda M Alvarez Costa, Manon Schensema, Salomé Zweekhorst, Lindy SwinkelsDiepenmaat, Stefanie Schipperen, Willem W van Kempen, Deborah Font Julia, Jan J C Jonker, Lauri Hanewinckel, Joyce Olsthoorn, Johan C Berends, Arie C van der Spek, Roy van der Berg, Rob J Timmermann, Ingrid Boerema, Iryna Mudruk, Anna Khrystoforova, Serhii Kyselov, Yaroslava V Hilova, Pavlo Logoida, Pavlo Logoida, Nataliia A Sanina, Ilona P Golikova, Olena O Nemchyna, Ilona P Golikova, Ilona P Golikova, Ivan I Isaichikov, Olga B Potapova, Iurii V Gura, Larysa Berestetska, Olena O Kulianda, Oleksandr Tantsura, Oleksandr S Kulbachuk, Volodymyr Petsentiy, Ihor Biskub, Ihor Biskub, Tetyana Handych, Oleg Lagkuti, Alyna Gagarina, Taras Chendey, Oksana F Bilonko, Olena Matova, Larysa Bezrodna, Olena Yarynkina, Tetiana Ovdiienko, Volodymyr Randchenko, Maryna Mospan, Tetiana Ovdiienko, Olena Butko, Olga Romanenko, Mykhailo Pavelko, Iryna Sichkaruk, Svitlana O Lazareva, Olena A Kudryk, Inessa M Koltsun, Inessa M Koltsun, Tetiana Magdalits, Sergei Zadorozhniy, Kira Kompaniiets, Andrii Ivanov, Sergiy Romanenko, Pavlo Kaplan, Vadym Y Romanov, Oksana P Mykytyuk, Nataliia S Zaitseva, Sergiy N Pyvovar, Lyudmyla Burdeuna, Emerita Serdobinska, Tatiana I Shevchenko, Igor I Ivanytskyi, Igor I Ivanytskyi, Igor I Ivanytskyi, Olena V Khyzhnyak, Ganna Smirnova, Nataliya Kalinkina, Olena Keting, Olena Sklyanna, Olga Kashanska, Anna Shevelok, Marina Khristichenko, Ievgenii Y Titov, Danilenko O Oleksander, Nataliia S Polenova, Nataliia Altunina, Viktoriia Kororaieva, Stanislav Zborovskiy, Leonid Kholopov, Iurii Suliman, Lanna Lukashenko, Stanislav Shvaykin, Olexandr M Glavatskiy, Roman O Sychov, Roman L Kulynych, Oleksandr A Skarzhevskyi, Nataliia V Dovgan, Marta Horbach, Olga Cherkasova, Iryna Tyshchenko, Liudmyla Todoriuk, Svitlana Kizim, Nataliia Brodi, Oleksandr Ivanko, Olga Garbarchuk, Liudmyla Alieksieieva, Tetiana L Shandra, Olena Beregova, Larisa An Bodretska, Svitlana S Naskalova, Ivanna A Antoniuk-Shcheglova, Olena V Bondarenko, Natalia G Andreeva, Iryna I Vakalyuk, Olha S Chovganyuk, Nataliya R Artemenko, Kiril A Maltsev, Natalia Kalishevich, Natalia G Kondratyeva, Svetlana A Nikitina, Maria V Martjanova, Anna V Sokolova, Dmitrii O Dragunov, Olga Kolesnik, Vera Larina, Oxana V Tsygankova, Maria Ivanova, Illia A Karpov, Elena M Aronova, Ekaterina S Vedernikova, Ekaterina I Lubinskaya, Taras Y Burak, Sergey I Skichko, Farhad Rasulev, Ekaterina B Soldatova, Alexander L Fenin, Ilya I Laptev, Elena E Luchinkina, Alexandr Akatov, Natalia V Polenova, Natalia N Slavina, Irina N Korovnika, Marina Yu Prochorova, Regina Shakirova, Elena N Andreicheva, Olga A Krasnova, Tinatin V Lobzhanidze, Tatiana B Dmitrova, Viktoriya V Stakhiv, Maria I Pechatnikova, Alexandra V Panova, Maria Y Tipikina, Oxana P Rotar, Nikolay A Bokovin, Saule K Karabalieva, Farid Y Tumarov, Elena V Vasileva, Natalya Gennadevna Lozhkina, Ekaterina V Filippova, Alisa I Sharkaeva, Ekanerina V Filippova (Deilik), Natalia Yu Tolkacheva, Elena N Domracheva, Andrey N Ryabikov, Inga T Abesadze, Marianna Z Alugishvili, Elena P Nikolaeva, Nadezda V Smirnova, Valentina I Rodionova, Polina V Dolovstaya, Igor E Yunonin, Sergey V Kadin, Tatyana S Sveklina, Anna V Bushmanova, Anna V Bushmanova, Elena L Barkova, Irina S Gomova, Yana V Brytkova, Tatiana B Ivanova, Marina Y Zubareva, Inga Skopets, Lybov A Galashevskaya, Emilia D Butinskaya, Olga G Gusarova, Natalia B Kalishevich, Yana R Pavlova, Marianna P Serebrenitskaya, Vitalina F Grygorieva, Gulnara R Kuchaeva, Inna A Vasileva, Gulnara I Ospanova, Yulia V Vahrusheva, Irina A Semenova, Irina E E Mikhailova, Olga O Kvasova, Valeria D Shurygina, Alexey E Rivin, Alexey O Savelyev, Alexey A Savelyev, Olesya O Milyaeva, Nadezhda N Lapshina, Ninel A Lantsova, Pavel V Alexandrov, Evgeniy A Orlikov, Alla Falkovskaya, Tatiana Ripp, Sergei Triss, Stanislav Pekarskiy, Sitkova Ekaterina, Evgeniya N Zhuravleva, Olga Perova, Galina Kovaleva, Liubov Koroleva, Liubov Koroleva, Lydia Mishchenko, Boris P Garshin, Svetlana A Kutuzova, Lyudmila I Provotorova, Igor P Zadvorny, Olga V Okhapkina, Anatoly O Khrustalev, Tatiana Suvorova, Elena S Shaf, Varvara A Vershinina, Andrey A Kozulin, Oxana A Oleynik, Irina Y Martynova, Natalia V Kizhvatova, Alla S Salasyuk, Vera V Tsoma, Alla A Ledyaeva, Elena V Chumachek, S C Blignaut, Tersia Y Alexander, Chano Du Plessis, Thirumani Govender, Samatha M Du Toit, Leya Motala, Areesh Gassiep, Christina Naude (Smit), Marli Terblanche, Marlien Snoer (Kruger), Berenice Pillay, De Vries Basson, Clive H Corbett, Marisa E Theron, Bianca Fouche, Mareli E Coetzee, Pieter Odendall, Frederik H Van Wijk, Anna-Mari Conradie, Trudie Van der Westhuizen, Carine Tredoux, Mohamed S Mookdam, Andie J Van der Merwe, Karin Snyman, Gerda Smal, Yvonne De Jager, Thomas A Mabin, Annusca King, Lindy L Henley, Brenda M Zwane, Jane Robinson, Marinda Karsten, Andonia M Page, Valerie Nsabiyumva, Charmaine Krahenbuhl, Jaiprakash D Patel, Yunus E Motala, Ayesha Dawood, Nondumiso B Koza, Lenore M S Peters, Shavashni Ramlachan, Wilhelm J Bodenstein, Pierre Roux, Lizelle Fouche, Cecilia M Boshoff, Haroon M Mitha, Fathima Khan, Henry P Cyster, Helen Cyster, E C Wessels, Florence J Jacobs, Melanie A Sebastian, Deborah A Sebastian, Nadia Mahomed, Ignatius P Immink, Celia Cotzee, Tanja Cronje, Madele Roscher, Maria Le Roux, Yvonne A Trinder, Renata Wnętrzak-Michalska, Magdalena Piszczek, Andrzej Piela, Ewa Czernecka, Dorota Knychas, Alina Walczak, Izabella Gładysz, Katarzyna Filas, Ewelina Kiluk, Krzysztof Świgło, Iwona Jędrzejczyk, Kamila Łuczyńska, Katarzyna Tymendorf, Wojciech Piesiewicz, Wojciech L Kinasz, Stefan Samborski, Ilona Bartuś, Gramzyna Latocha Korecka, Ewa Gulaj, Jolanta Sopa, Bogusław Derlaga, Marcin Baisiak, Allicia Kowalisko, Edyta Stainszewska-Marasazlek, Bartosz Szafran, Malgorzata Swiatkiewicz, Artur Racewicz, Sławomir Grycel, Jerzy Supronik, Sylwia Walendziuk, Magdalena Tarantowicz, Agata Stasiak, Anna Sidorowicz-Białynicka, Marek Dwojak, Ewa Jaźwińska-Tarnawska, Katarzyna Kupczyk, Kamila Martowska, Kamila Kulon, Katarzyna Gajda, Bivin Wilson, Krithika Velusamy, Swaidha S Sadhiq, Bhavani Siddeshi, M Bhanukumar, Abhishek Srivatsav, Madhan Ramesh, Sri Harsha Chalasani, Mini Johnson, Prashanth Gopu, Jeesa George, Sowmya Reddy, Swetha Tessy Thara Eleena, Damodara Rao Kodem, Haritha N Nakkella, Padma Kumari Mandula, Anjan Kumar Vuriya, Syamala Rajana, Aruna Kale, Tiwari Rajeev, Raina Jain, Vipin Jain, Srilakshmi Mandayam Adhyapak, Lumin Sheeba, C R Uma, R Ramya, Aditya V Kulkarni, M S Ganachari, Ruma Sambrekar, Mohammad Bilal, Nungshijungla, Kalyan Chakravarthy, Ravi Badhavath, Sravan Kumar, Meenakshi Simhadri, Farooque Salamuddin, Venkat Prasad, Vivek Dwivedi, Sudha Sarna, Tilak Arora, Deepak Chawla, Archana Sathe, Chaware Gayatree, Ajeet Nanda, Ram Avtar, Jyoti Sharma, Vaibhavi P S Sasirekha D, Deepthi Kobbajji, Ramya Ningappa, Shwetha Shree, K Chandrashekar, M R Nandini, S Sowjanya, I G Devika, N Yashaswini, G Sonika, L Rathna, R Priyanka, Rupal J Shrimanker, Lakshmi Vinutha Reddy, K Sumathi, Babitha Devi, Bina N Naik, Rohini Manjunath, Rajeshwari Ashok, Tony V Kunjumon, Jesline Thomas, Shaik Samdhani, Kasthuri Selvam, Poongothai Subramani, Nandakumar Parthasarathy, Nirmal K Bohra, Anvesh K Gatla, Cheryl Horbatuk, Julie Sills, E B Davey, Liz Paramonczyk, Olga Racanelli, David Crowley, Sandy Strybosch, Andre Belanger, Jean Palardy, Alicia Schiffrin, Sylvie Gauthier, Norman Kalyniuk, Shawn D Whatley, Heather Lappala, Grishma Patel, Matthew Reeve, Catherine Moran, Jody Everitt, Teresa Ferrari, Christine Bouffard, Jirir Frohlich, Gordon Francis, John Mancini, Gregory Bondy, Debbie DeAngelis, Patricia Fulton, Debbie DeAngelis, Patricia Fulton, David W Blank, Angela Lombardo, Mylène Roy, Jackie Chow, Hyman Fox, William J Grootendorst, Angela Hutchinson, Hyman Fox, Sharon M Chan, Christie Fitzgerald, Teresa Ferrari, Lynn Wilkins, Rebecca L Raymond, Arlene Reyes, Lavoie Marc André, Denis Fortin, Hélène Ouimet, Thanh-Thao Tôn-Nu, Martine Dussureault, Marie-Hélène Blain, Madeleine Roy, Nathalie Kopajko, Chantal Fleury, Karine Maheux, Gabriela Valentina Ciobotaru, Maria C Constantinescu, Carmen-Lucia Gherghinescu, Ana-Maria Avram, Ioan Manitiu, Radu I Cojan, Octavian M Pirvu, Aura Sinpetrean, Lucian Pop, Delia Lupu, Radu Usvat, Ana Petrisor, Nicoleta Dumitru, Camelia Moruju, Adelina Gheorghita, Magda V Mitu, Cosmin Macarie, Ana Maria Pop, Maria-Catalina Diaconu, Iulia Grancea, Mihaela Cosma, Mihaela Cosma, Mihaela Crisan, Elizabeth Herron, Anthony M Dart, Paul Nestel, Sally B Kay, Kaye S Carter, Imran Badshah, Ashley Makepeace, Jocelyn Drinkwater, Michelle England, Azette Rafei, Kylie Patterson, Alicia Jenkins, Sybil McAuley, Sue M Kent, Joy E Vibert, Leonie Perrett, Thomas David, Samantha L Kaye, Monika O'Connor, Nimalie J Perera, Nicole T Lai, Kerry A Kearins, Christinia Dicamillo, Heather Anderson, Louise Ferguson, Sharon D Radtke, Charles T Thamarappillil, Janice M Boys, Anita K Long, Toni Shanahan, Michael Nyguyen, Nicole Forrest, Gill Tulloch, Della Greenwell, Sarah L Price, Aye N Tint, Priya K Sumithran, Tamara L Debreceni, Lisa Walker, Mary Caruana, Kira Edwards, Maria Stathopoulos, Cilla Haywood, Dimitar Sajkov, Sharen Pringle, Anne Tabner, Kathrina Bartolay, Chamindi Abeyratne, Kylie Bragg, Patrick Mulhern, Peter Purnell, Randall Hendriks, Gill Tulloch, Lyn Williams, Jane Hamlyn, Aurelia Connelly, Jan Hoffman, Samantha Bailey, Jane Kerr, Zarnia Morrison, Sarah Maeder, Roberta McEwan, Prasanna Kunasekera, Patrice McGregor, Jo Young, Sharon Berry, Rick Cutfield, Michelle Choe, Catherine McNamara, Narrinder K Shergill, Petra Crone, Miles G Williams, Keith Dyson, Diana H Schmid, Audrey C Doak, Melissa Spooner, Colin Edwards, Anne Turner, Grainne M McAnnalley, Raewyn A Fisher, Fraser B Hamilton, Denis H Friedlander, Melissa R Kirk, Jayne E Scales, Marguerite A McLelland, Neelam A Dalman, Cathy E Vickers, Carolyn Jackson, Wendy Coleman, Phillip I Garden, Wendy F Arnold

**Affiliations:** Department of Vascular Medicine, University Medical Centre Utrecht, Utrecht 3584 CX, the Netherlands; Mount Sinai Heart, Icahn School of Medicine at Mount Sinai Health System, NY 10029, USA; Department of Vascular Medicine, University Medical Centre Utrecht, Utrecht 3584 CX, the Netherlands; Dutch Cardiovascular Research Network (WCN), Utrecht 3511 EP, the Netherlands; Department of Cardiology, Green Heart Hospital, Gouda 2803 HH, the Netherlands; Dutch Cardiovascular Research Network (WCN), Utrecht 3511 EP, the Netherlands; Department of Cardiology, Meander Medical Centre, Amersfoort 3813 TZ, the Netherlands; Dutch Cardiovascular Research Network (WCN), Utrecht 3511 EP, the Netherlands; Department of Cardiology, Amsterdam University Medical Centre, Amsterdam 1105 AZ, the Netherlands; Université Paris-Cité, LVTS, French Alliance for Cardiovascular Trials (FACT), Assistance Publique-Hôpitaux de Paris, Paris 75018, France; Department of Vascular Medicine, University Medical Centre Utrecht, Utrecht 3584 CX, the Netherlands

**Keywords:** Icosapent ethyl, Eicosapentaenoic acid, Triglycerides, Atherosclerotic cardiovascular disease, Residual risk, Secondary prevention

## Abstract

**Aims:**

Icosapent ethyl lowers triglycerides and significantly reduces major adverse cardiovascular events (MACE), though treatment effects may vary between individuals. This study aimed to determine the relative and absolute effects of icosapent ethyl on MACE according to baseline cardiovascular disease (CVD) risk in patients with atherosclerotic cardiovascular disease (ASCVD).

**Methods and Results:**

Participants from the Reduction of Cardiovascular Events with Icosapent Ethyl—Intervention Trial (REDUCE-IT) with ASCVD were included (*n* = 5785). The primary outcome was 3-point MACE, i.e. non-fatal myocardial infarction, non-fatal stroke, or cardiovascular death. Baseline 5-year risk of MACE was estimated using the European Society of Cardiology (ESC) guideline-recommended SMART2 risk score. Modification of the relative treatment effects of icosapent ethyl by baseline risk was assessed using Cox proportional hazards models, including a treatment-by-risk interaction. Next, treatment effects were assessed stratified by quartiles of baseline risk. During a median follow-up of 4.8 years (interquartile range 3.2–5.3), MACE occurred in 361 vs. 489 patients in the icosapent ethyl vs. placebo group [95% confidence interval (CI)]; hazard ratio (HR) 0.72 (0.63–0.82), absolute risk reduction (ARR) 4.4% (2.6–6.2%), number needed to treat (NNT) 23 (16–38), and 5-year Kaplan-Meier estimated cumulative incidence reduction (CIR) 5.7% (3.5–7.9%). Icosapent ethyl significantly reduced MACE in all risk quartiles, with an HR (95% CI) of 0.62 (0.43–0.88), 0.66 (0.48–0.92), 0.69 (0.53–0.90), and 0.78 (0.63–0.96), respectively (*P* for treatment-by-risk interaction = 0.106). The ARR (95% CI) increased across risk quartiles, i.e. was 3.9% (1.0–6.8%), 4.3% (1.2–7.3%), 5.1% (1.4–8.7%), and 5.6% (1.3–10.0%), respectively. This translates to NNTs (95% CI) of 26 (15–98), 24 (14–84), 20 (11–70), and 18 (10–77). The 5-year CIR (95% CI) was 4.8% (1.3–8.2%), 5.0% (1.3–8.7%), 6.1% (1.7–10.5%), and 7.7% (2.3–13.2%), respectively. Consistent results were obtained for 5-point MACE, additionally including coronary revascularization and unstable angina.

**Conclusion:**

Among patients with ASCVD and elevated triglyceride levels, icosapent ethyl significantly reduces the risk of MACE irrespective of baseline CVD risk, though absolute benefits are largest for patients at the highest risk.

## Graphical Abstract

**Graphical Abstract:**
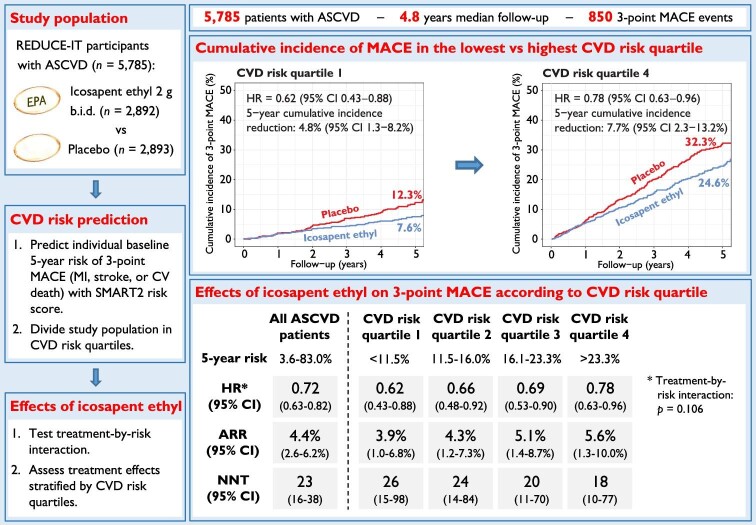

## Introduction

Patients with atherosclerotic cardiovascular disease (ASCVD) remain at high risk of recurrent cardiovascular events, despite the routine use of lipid-lowering, blood pressure-lowering, and antithrombotic therapies.^1,2^ This residual risk can be partially attributed to elevated triglyceride levels.^3,4^ In the Reduction of Cardiovascular Events with Icosapent Ethyl—Intervention Trial (REDUCE-IT), icosapent ethyl, a highly purified eicosapentaenoic acid (EPA) ethyl ester that lowers triglycerides, reduced the risk of major adverse cardiovascular events (MACE) by ∼25%.^5^ Clinical guidelines state that icosapent ethyl may be considered to reduce residual cardiovascular disease (CVD) risk in high-risk patients with ASCVD and triglycerides >1.5/>1.7 mg/dL (>135/>150 mmol/L) despite optimal statin treatment.^6–10^

In clinical trials such as REDUCE-IT, the treatment effect is usually reported on a group level in terms of an average hazard ratio (HR). However, considerable differences in treatment efficacy may exist between individuals.^11,12^ For example, treatment effects may differ between patients with and without a history of ASCVD. The absolute effects of icosapent ethyl in patients with established ASCVD specifically, have not been assessed yet. Also, within patients with ASCVD, there may be heterogeneity of treatment effects. First, the relative treatment effect (i.e. the HR) may be modified by a patient's characteristics or baseline risk of CVD. Second, even in case of an equal relative treatment effect, the absolute treatment effect [i.e. absolute risk reduction (ARR) or gain in CVD-free survival] may still vary substantially between patients based on differences in baseline CVD risk and remaining life expectancy. Previous reports have proposed that this heterogeneity of treatment effects should be assessed systematically in all trials by evaluating the interaction between treatment effects and individual baseline risk as predicted by a multivariable risk model.^11,13,14^ The SMART2 and SMART-REACH risk models are the European Society of Cardiology (ESC) guideline-recommended tools for prediction of 10-year and lifetime CVD risk in patients with ASCVD.^6,15,16^ Applying these models to REDUCE-IT participants and evaluating the impact of baseline CVD risk on the efficacy of icosapent ethyl could help identify the optimal target population for icosapent ethyl therapy, which could support individualized clinical decision making and future guideline recommendations.

This study aimed to assess the relative and absolute treatment effects of icosapent ethyl on MACE according to individual baseline CVD risk in patients with ASCVD.

## Methods

### Study population

REDUCE-IT was a randomized, double-blind, placebo-controlled trial (registration number: NCT01492361) in which participants were randomly assigned to receive 2 g of icosapent ethyl twice daily or placebo.^5^ Patients were eligible if they had established ASCVD or diabetes mellitus with at least one additional risk factor, had a fasting triglyceride level of 150–499 mg/dL (1.69–5.63 mmol/L) and a low-density lipoprotein cholesterol (LDL-c) level of 41–100 mg/dL (1.06–2.59 mmol/L), and had been receiving a stable dose of a statin for at least 4 weeks (complete eligibility criteria in Supplementary material online, *Table S1*). Detailed descriptions of the trial have been published elsewhere.^5,17^ The trial was approved by the local health authorities, institutional review boards, and ethics committees, and written informed consent was obtained from all participants. For the current study, all participants with established ASCVD (*n* = 5785) were selected.

### SMART2 and SMART-REACH risk models

The SMART2 and SMART-REACH risk models are the ESC guideline-recommended tools for prediction of 10-year (SMART2) and lifetime (SMART-REACH) risk of CVD [i.e. non-fatal myocardial infarction (MI), non-fatal stroke, or cardiovascular death] in patients with ASCVD.^6,15,16^ For the current study, SMART2 was used to predict 5-year CVD risk using the 5-year baseline survival provided in the original report to be able to directly validate the predicted risks to the observed risks in REDUCE-IT (median follow-up ∼5 years).^15^ Predictions are based on a selection of established CVD risk factors (Supplementary material online, *Table S2*). Descriptions of the development and validation of the models have been published previously.^15,16^ Online calculators are freely available on www.U-Prevent.com.

### Outcomes

The primary outcome was 3-point MACE, i.e. a composite of non-fatal MI, non-fatal stroke, or cardiovascular death. Three-point MACE was chosen as the primary outcome, as this is the outcome originally predicted by the SMART2 and SMART-REACH models as recommended by ESC guidelines.^6^ The secondary outcome was 5-point MACE, i.e. a composite of non-fatal MI, non-fatal stroke, cardiovascular death, coronary revascularization, or unstable angina (the primary endpoint of REDUCE-IT). Detailed outcome definitions are provided in Supplementary material online, *Table S3*.

### Statistical analysis

#### Efficacy of icosapent ethyl in patients with ASCVD

Hazard ratios for the relative treatment effect of icosapent ethyl on 3-point MACE, 5-point MACE, and their components were established using Cox proportional hazards models. The ARR was calculated as the proportion of patients with an event in the placebo group minus the proportion of patients with an event in the icosapent ethyl group at the end of follow-up. The number needed to treat (NNT) was calculated as 1/ARR. In addition, reductions in the cumulative incidence of MACE were calculated as the difference between the Kaplan-Meier estimated cumulative incidence of MACE in the placebo vs. icosapent ethyl group at 5-year follow-up, i.e. the 5-year cumulative incidence reduction (CIR). Advantages of this method over the conventional ARR calculation are that it takes the time to event and censoring into account and has a clear timespan (5 years in this case). The conventional ARR was still reported as well to allow a comparison between this study and the main trial report or previous REDUCE-IT subgroup analyses.^5,18–24^

#### Validation of the SMART2 and SMART-REACH risk models in REDUCE-IT

The SMART2 and SMART-REACH risk models were externally validated in REDUCE-IT. The models were recalibrated to match the underlying event rates in the study population (Methods S1). Model performance was assessed using c-statistics for discrimination and plots of the predicted vs. observed 5-year risk for calibration. The SMART2 risk score was then used to estimate the baseline 5-year risk of 3-point MACE for all individuals in the study population. Based on these estimates, the population was divided into risk quartiles. Baseline characteristics were presented stratified by these quartiles.

#### Interaction between baseline 5-year CVD risk and the treatment effects of icosapent ethyl

To assess whether the relative treatment effect of icosapent ethyl was modified by baseline CVD risk, a Cox proportional hazards model was derived for 3-point MACE, with allocation to icosapent ethyl, predicted baseline 5-year risk, and an interaction between these two as predictors, in line with previously proposed methods.^11,13,14^ The treatment-by-risk interaction was tested for statistical significance using a likelihood ratio test comparing a model with to a model without the interaction term. Next, the relative treatment effect of icosapent ethyl was determined within each risk quartile by deriving quartile-specific models. Heterogeneity of absolute treatment effects was assessed by calculating the ARR, NNT, and 5-year CIR in each quartile separately, using the same methods applied in the overall study population.

#### Lifetime CVD risk and treatment benefits of icosapent ethyl

The SMART-REACH model was used to estimate the baseline lifetime risk of 3-point MACE and MACE-free survival for all individuals in the study population. The lifetime benefits of icosapent ethyl were estimated by combining the model with the average relative treatment effect for patients with ASCVD presented in the original trial report, i.e. an HR of 0.72, according to previously described methods (explained in Methods S2).^12,16^ MACE-free survival with and without icosapent ethyl was presented for each risk quartile in survival curves based on the average predicted survival in each quartile at 1-year time intervals. Lifetime benefit was expressed in terms of MACE-free life-years gained, defined as the difference between the median MACE-free survival (where the curve crosses 50%) with and without icosapent ethyl (Methods S2).

#### Continuous relation between baseline CVD risk and the treatment effects of icosapent ethyl

In addition to the analyses in risk quartiles, the relation between baseline 5-year risk and the treatment effects of icosapent ethyl was also assessed continuously. For the relative treatment effect, this was done by deriving a Cox model including a restricted cubic splines function for the treatment-by-risk interaction. As the ARR and 5-year CIR cannot be directly derived from a statistical model, these were determined in increasingly small risk groups, after which their continuous relation with baseline risk was estimated using a restricted cubic splines function weighted for the accuracy of the estimate in each group. Corresponding 95% confidence intervals (CIs) were derived from 10 000 bootstrap samples. A detailed description of the methodology is provided in Methods S3.

The same analyses were performed for 5-point MACE. To be able to predict the risk of 5-point MACE, even though the models were originally developed for 3-point MACE, the models’ baseline risks were recalibrated to the event rate of this new outcome while using the original model coefficients (Methods S1). As this only changes the absolute risk value, rather than the ranking of participants, the distribution of participants over the risk quartiles was the same for 3-point and 5-point MACE.

Missing data [≤0.3% for all predictor variables except years since the first CVD event (9.9%); Supplementary  *Table S4*] were imputed by single imputation using predictive mean matching. All analyses were conducted with R statistical software V.4.0.3 (www.r-project.org).

## Results

### Baseline characteristics

Baseline characteristics are presented for the total ASCVD population and stratified for CVD risk quartiles in *Table 1*. Predicted baseline 5-year risk of 3-point MACE across risk quartiles was [mean (range)]: 9.0% (3.6–11.4%), 13.7% (11.5–16.0%), 19.3% (16.1–23.3%), and 33.7% (23.4–83.0%), and the risk of 5-point MACE was: 14.3% (5.8–18.0%), 21.5% (18.1–24.9%), 29.7% (25.0–35.3%), and 48.4% (35.4–94.6%). Levels of non-lipid CVD risk factors increased across risk quartiles, while lipid concentrations and the use of lipid-lowering therapy remained relatively stable (*Table 1*).

**Table 1:** Baseline characteristics

	Total ASCVD population (*n* = 5785)	Risk quartile 1 (*n* = 1452)	Risk quartile 2 (*n* = 1442)	Risk quartile 3 (*n* = 1445)	Risk quartile 4 (*n* = 1446)
**SMART2 predicted 5-year risk** ^a^					
3-point MACE (%), mean (range)	18.9 (3.6–83.0)	9.0 (3.6–11.4)	13.7 (11.5–16.0)	19.3 (16.1–23.3)	33.7 (23.4–83.0)
5-point MACE (%), mean (range)	28.4 (5.8–94.6)	14.3 (5.8–18.0)	21.5 (18.1–24.9)	29.7 (25.0–35.3)	48.4 (35.4–94.6)
**Demographic**					
Age	63.2 ± 8.7	56.1 ± 6.5	60.9 ± 6.9	65.2 ± 6.7	70.8 ± 7.0
Sex (male)	4536 (78%)	1109 (76%)	1128 (78%)	1120 (78%)	1179 (82%)
Geographic region					
United States and Canada	2010 (35%)	380 (26%)	443 (31%)	514 (36%)	673 (47%)
The Netherlands	1499 (26%)	338 (23%)	378 (26%)	397 (28%)	386 (27%)
Eastern Europe	1667 (29%)	553 (38%)	455 (32%)	390 (27%)	269 (19%)
Australia and New Zealand	234 (4%)	71 (5%)	65 (5%)	52 (4%)	46 (3%)
Other^b^	375 (6%)	110 (8%)	101 (7%)	92 (6%)	72 (5%)
Current smoking	956 (17%)	101 (7%)	239 (17%)	281 (19%)	335 (23%)
**History of ASCVD**					
Coronary artery disease	4532 (78%)	1057 (73%)	1128 (78%)	1160 (80%)	1187 (82%)
Cerebrovascular disease	1147 (20%)	98 (7%)	180 (13%)	309 (21%)	560 (39%)
Peripheral artery disease	688 (12%)	56 (4%)	109 (8%)	179 (12%)	344 (24%)
Abdominal aortic aneurysm	75 (1%)	0 (0%)	3 (0%)	13 (1%)	59 (4%)
Years since first manifestation of ASCVD, median (IQR)	6 (2–11)	2 (1–5)	4 (1–9)	7 (3–12)	10 (5–16)
**Comorbidities**					
Diabetes mellitus	2401 (42%)	236 (16%)	516 (36%)	703 (49%)	946 (65%)
Atrial fibrillation	550 (10%)	66 (5%)	110 (8%)	140 (10%)	234 (16%)
Heart failure	1215 (21%)	305 (21%)	278 (19%)	291 (20%)	341 (24%)
**Physical examination**					
Body-mass index (kg/m^2^)	31.0 ± 5.0	30.6 ± 4.7	30.9 ± 4.9	31.2 ± 4.9	31.0 ± 4.9
Systolic blood pressure (mmHg)	133 ± 16	129 ± 13	132 ± 15	134 ± 15	136 ± 17
**Laboratory measurements**					
Non-HDL-cholesterol (mmol/L)	3.1 ± 0.5	3.0 ± 0.5	3.1 ± 0.5	3.1 ± 0.5	3.2 ± 0.5
LDL-cholesterol (mmol/L)	2.0 ± 0.5	1.9 ± 0.5	2.0 ± 0.5	2.0 ± 0.5	2.0 ± 0.5
HDL-cholesterol (mmol/L)	1.0 ± 0.2	1.1 ± 0.2	1.1 ± 0.2	1.0 ± 0.2	1.0 ± 0.2
Triglycerides (mmol/L)	2.7 ± 0.9	2.5 ± 0.8	2.6 ± 0.9	2.7 ± 0.9	2.7 ± 0.9
Estimated GFR (mL/min/1.73 m^2^)	74 ± 18	84 ± 13	79 ± 15	72 ± 16	61 ± 17
C-reactive protein (mg/L), median (IQR)	2.0 (1.0–4.2)	1.3 (0.7–2.4)	2.1 (1.0–3.7)	2.4 (1.2–4.6)	3.1 (1.5–6.2)
Eicosapentaenoic acid (µg/mL), median (IQR)	27.2 (17.2–41.7)	27.6 (17.2–42.6)	27.3 (17.2–41.3)	27.4 (17.6–43.7)	26.1 (16.9–40.0)
**Medication use**					
Allocation to icosapent ethyl	2892 (50%)	757 (52%)	699 (48%)	694 (48%)	742 (51%)
Statin intensity					
Low	223 (4%)	32 (2%)	49 (3%)	60 (4%)	82 (6%)
Moderate	3520 (61%)	869 (60%)	861 (60%)	886 (61%)	904 (63%)
High	2026 (35%)	549 (38%)	528 (37%)	495 (34%)	454 (31%)
Ezetimibe	431 (7%)	98 (7%)	106 (7%)	115 (8%)	112 (8%)
Antihypertensive agents					
None	212 (4%)	68 (5%)	52 (4%)	57 (4%)	35 (2%)
One	1065 (18%)	355 (24%)	286 (20%)	240 (17%)	184 (13%)
Two	2176 (38%)	649 (45%)	583 (40%)	522 (36%)	442 (29%)
Three or more	2332 (40%)	380 (26%)	521 (36%)	626 (43%)	805 (56%)
Antithrombotic therapy					
None	296 (5%)	42 (3%)	62 (4%)	90 (6%)	102 (7%)
Antiplatelet monotherapy	3566 (62%)	873 (60%)	934 (65%)	906 (63%)	853 (59%)
Dual antiplatelet therapy	1574 (27%)	508 (35%)	384 (27%)	344 (24%)	338 (23%)
Vitamin K antagonist	505 (9%)	49 (3%)	88 (6%)	138 (10%)	230 (16%)

All data in *n* (%) or mean ± SD, unless otherwise specified.

ASCVD, atherosclerotic cardiovascular disease; GFR, glomerular filtration rate; HDL, high-density lipoprotein; IQR, interquartile range; LDL, low-density lipoprotein; MACE, major adverse cardiovascular events; SD, standard deviation.

a Baseline 5-year risk of MACE as predicted by the SMART2 risk score. For both 3-point and 5-point MACE the risks have been recalibrated to match the event rates for these outcomes in the study population.

b This includes India and South Africa.

### Efficacy of icosapent ethyl in patients with ASCVD

Three-point MACE occurred in 361 (12.5%) vs. 489 (16.9%) patients (HR 0.72; 95% CI 0.63–0.82), and 5-point MACE in 559 (19.3%) vs. 738 (25.5%) patients (HR 0.73; 95% CI 0.65–0.81) in the icosapent ethyl and placebo groups, respectively. Over a median follow-up of 4.8 years [interquartile range (IQR) 3.2–5.3], the ARR and NNT (95% CI) were 4.4% (2.6–6.2%) and 23 (16–38) for 3-point MACE and 6.2% (4.0–8.3%) and 16 (12–25) for 5-point MACE. The reduction in the Kaplan-Meier estimated cumulative incidence at 5 years, i.e. 5-year CIR (95% CI), was 5.7% (3.5–7.9%) for 3-point MACE, and 7.5% (5.0–10.0%) for 5-point MACE (Supplementary material online, *Figure S1*). Icosapent ethyl significantly reduced the risk of all other outcomes, except for fatal or non-fatal stroke (HR 0.78; 95% CI 0.58–1.05) and death from any cause (HR 0.86; 95% CI 0.72–1.04) (Supplementary material online, *Figure S1*).

### Performance of the SMART2 and SMART-REACH risk models in REDUCE-IT

**Figure 1:**
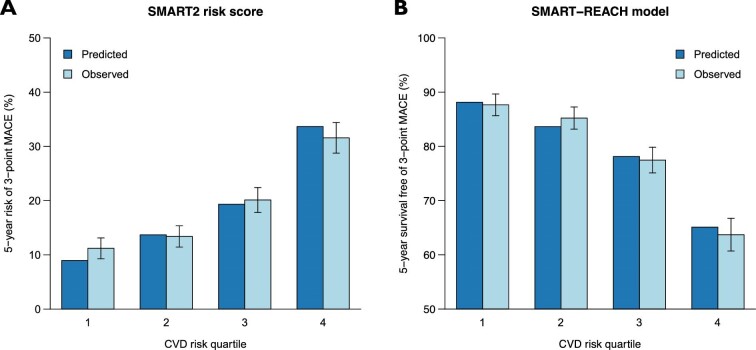
Calibration of the SMART2 and SMART-REACH risk models across CVD risk quartiles. Mean predicted (by the SMART2 risk score) vs. observed 5-year risk of 3-point MACE (*A*), and mean predicted (by the SMART-REACH model) vs. observed 5-year survival free of 3-point MACE (*B*) across CVD risk quartiles. Error bars represent 95% confidence intervals. Abbreviations: CVD, cardiovascular disease; MACE, major adverse cardiovascular events.

The predicted 5-year risk of MACE and survival free of MACE showed good agreement with the observed risk and survival in the CVD risk quartiles (3-point MACE in *Figure 1*; 5-point MACE in Supplementary material online, *Figure S2*). Consistent results were obtained when the models were validated in octiles of risk (Supplementary material online, *Figure S3*).

### Interaction between baseline CVD risk and the effects of icosapent ethyl

**Figure 2:**
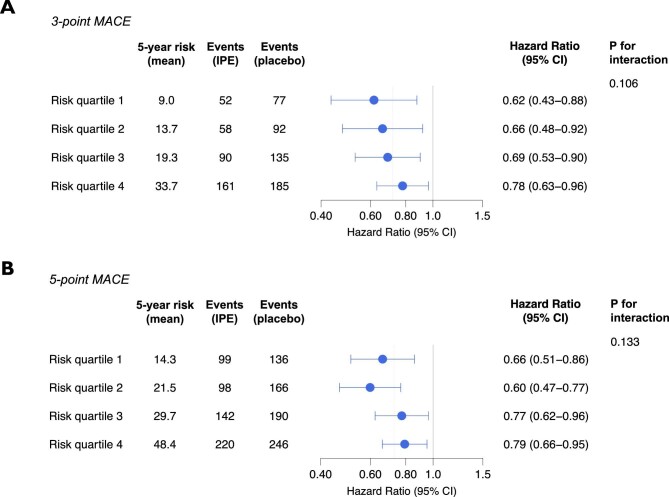
Relative treatment effects of icosapent ethyl across CVD risk quartiles. Relative treatment effects of icosapent ethyl on the risk of 3-point MACE (*A*) and 5-point MACE (*B*) in the CVD risk quartiles. The *P*-value is for the interaction between baseline risk and the relative treatment effect of icosapent ethyl. The grey dotted line denotes the overall trial hazard ratio. Abbreviations: CVD, cardiovascular disease; MACE, major adverse cardiovascular events.

**Figure 3:**
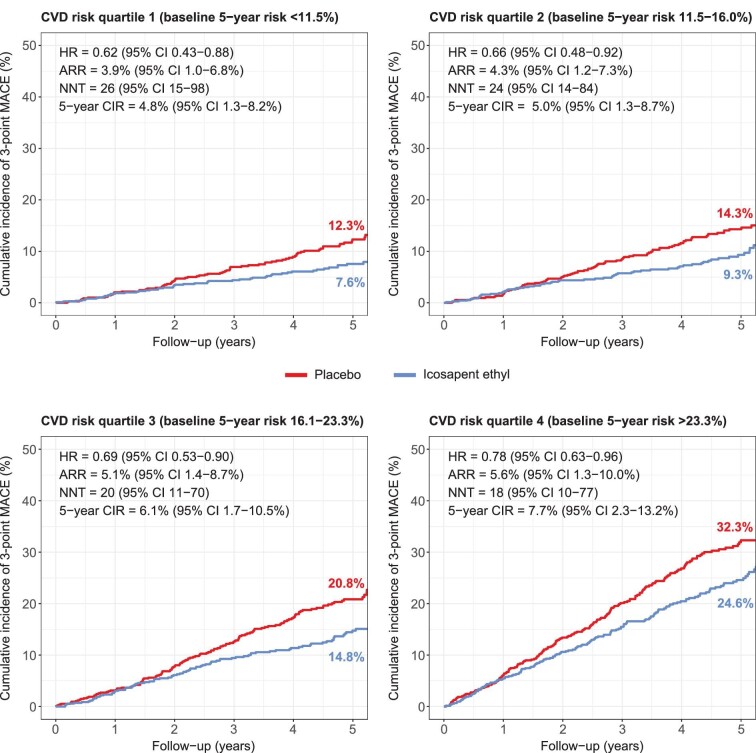
Absolute treatment effects of icosapent ethyl on 3-point MACE across CVD quartiles. Kaplan-Meier curves of the cumulative incidence of 3-point MACE in participants randomized to icosapent ethyl and placebo within each CVD risk quartile. Abbreviations: CVD, cardiovascular disease; MACE, major adverse cardiovascular events.

**Figure 4:**
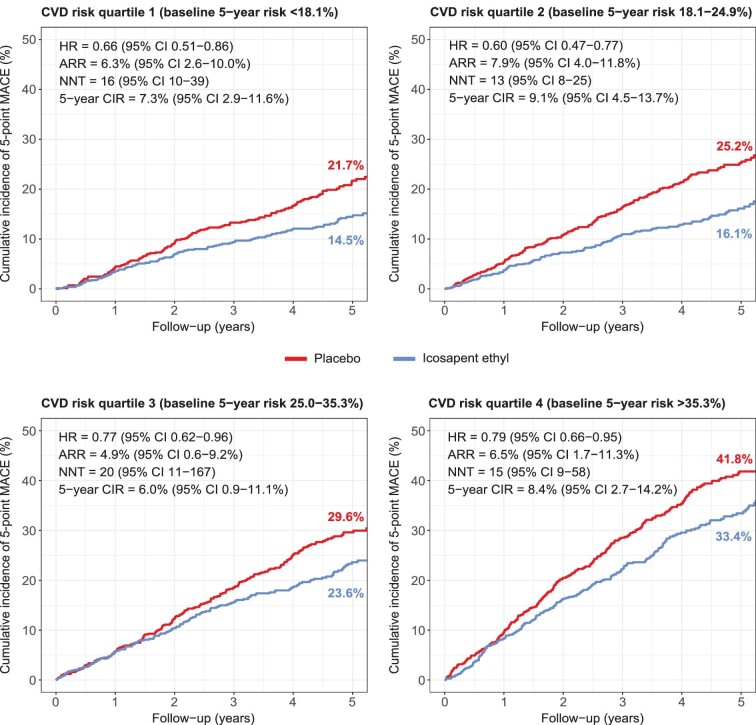
Absolute treatment effects of icosapent ethyl on 5-point MACE across CVD risk quartiles. Kaplan-Meier curves of the cumulative incidence of 5-point MACE in participants randomized to icosapent ethyl and placebo within each CVD risk quartile. Abbreviations: CVD, cardiovascular disease; MACE, major adverse cardiovascular events.

There was no significant interaction between baseline risk and the relative treatment effects of icosapent ethyl (*P* = 0.106 for 3-point MACE; *P* = 0.133 for 5-point MACE), although there was a non-significant trend towards an attenuation of the relative effect with increasing baseline risk (*Figure 2* & Central Illustration). The HR (95% CI) ranged from 0.62 (0.43–0.88) in the lowest to 0.78 (0.63–0.96) in the highest risk quartile for 3-point MACE, and from 0.66 (0.51–0.86) to 0.79 (0.66–0.95) for 5-point MACE. Despite the slight attenuation of the relative treatment effect, for 3-point MACE, the absolute treatment effects of icosapent ethyl increased with increasing baseline risk (*Figure 3*). The ARR and NNT (95% CI) ranged from 3.9% (1.0–6.8%) and 26 (15–98) in the lowest to 5.6% (1.3–10.0%) and 18 (10–77) in the highest risk quartile, and the 5-year CIR ranged from 4.8% (1.3–8.2%) to 7.7% (2.3–13.2%). For 5-point MACE, the favourable relative treatment effects in the lowest risk quartiles were accompanied by relatively large ARRs [6.3% (2.6–10.0%) in quartile 1; 7.9% (4.0–11.8%) in quartile 2], comparable to that observed in the highest risk quartile [6.5% (1.7–11.3%)] (*Figure 4*).

### Lifetime benefit from icosapent ethyl

**Figure 5:**
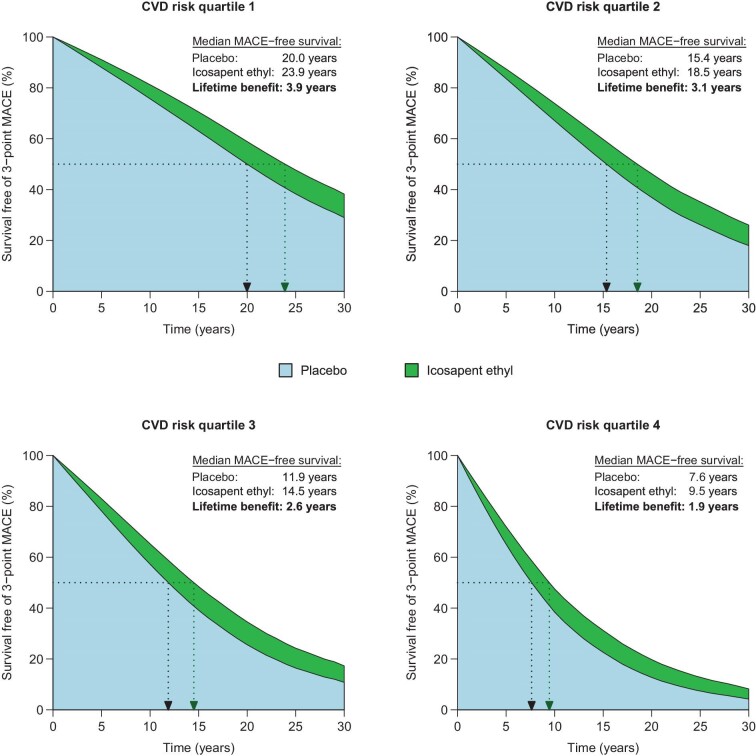
Lifetime benefit from icosapent ethyl across CVD risk quartiles. Average predicted survival free of 3-point MACE on placebo and on icosapent ethyl within each CVD risk quartile. Abbreviations: CVD, cardiovascular disease; MACE, major adverse cardiovascular events.

Predicted baseline survival free of 3-point MACE across risk quartiles was [median (IQR)]: 20.0 (10.3–32.6), 15.4 (7.6–25.7), 11.9 (5.7–20.3), and 7.6 (3.5–14.1) years, and survival free of 5-point MACE was: 14.7 (6.9–24.4), 11.0 (5.1–19.0), 8.4 (3.9–14.9), and 5.3 (2.3–9.8) years. The absolute lifetime benefit from icosapent ethyl, expressed as additional life years without MACE gained, decreased with increasing baseline risk. Gains in MACE-free survival ranged from 3.9 years (IQR 3.4–4.4) in the lowest to 1.9 years (IQR 1.5–2.2) in the highest risk quartile for 3-point MACE (*Figure 5*), and from 3.3 years (IQR 2.9–3.8) to 1.4 years (IQR 1.1–1.8) for 5-point MACE (Supplementary material online, *Figure S4*).

### Continuous relation between baseline CVD risk and the effects of icosapent ethyl

**Figure 6:**
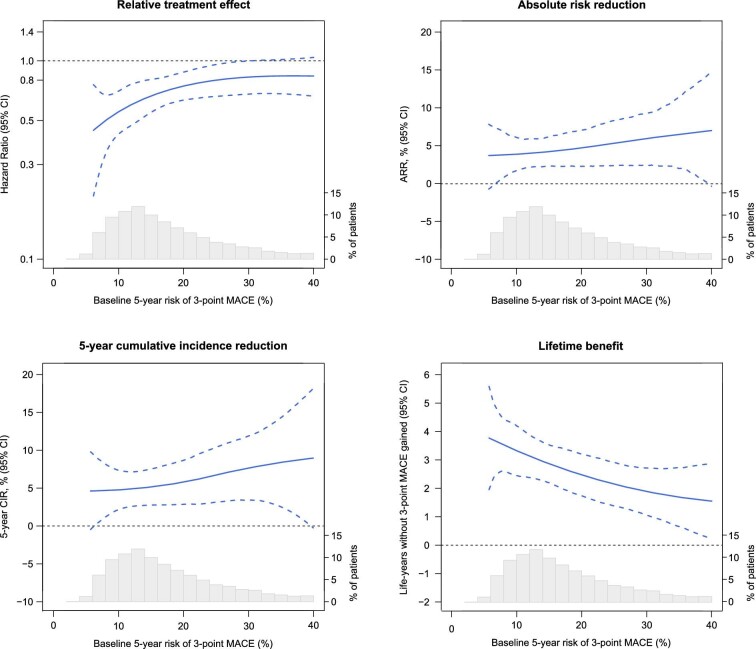
Continuous relation between baseline risk and the effects of icosapent ethyl. The continuous relation between baseline 5-year risk of 3-point MACE and the relative and absolute treatment effects of icosapent ethyl on the risk of 3-point MACE, derived from restricted cubic spline functions. The dotted lines denote 95% confidence intervals. The histogram shows the distribution of baseline risk in the study population Abbreviation: MACE, major adverse cardiovascular events.

For 3-point MACE, the HR (i.e. relative treatment effect) of icosapent ethyl was most favourable (∼0.60) in patients with the lowest baseline risk (5-year risk of 3-point MACE <10%), then gradually increased to ∼0.80 at a baseline risk of 20%, and remained largely stable thereafter (*Figure 6*). Despite the numerical attenuation of the relative treatment effect, the ARR and 5-year CIR gradually increased with increasing baseline risk. Absolute gains in MACE-free survival decreased with increasing baseline risk. Similar trends were observed for 5-point MACE (Supplementary material online, *Figure S5*).

## Discussion

Using data of 5785 participants from the REDUCE-IT trial, this study established the efficacy of icosapent ethyl in patients with ASCVD as well as demonstrated the heterogeneity of its relative and absolute treatment effects according to baseline CVD risk in this population (Central Illustration). Icosapent ethyl led to significant relative reductions in the risk of MACE across all CVD risk quartiles. There was no significant interaction between baseline risk and the relative treatment effect of icosapent ethyl, although there was a non-significant trend towards an attenuation of the relative effect with increasing baseline risk. Due to the favourable relative treatment effect at the lower end of the risk spectrum (HR ∼0.60), absolute treatment effects were already substantial (ARR ∼4%; 5-year CIR ∼5%) for patients in the lowest quartile of baseline CVD risk. Despite the slight numerical attenuation of the relative treatment effect towards the upper end of the spectrum (HR ∼0.80), absolute treatment effects were still the largest (ARR ∼6%; 5-year CIR ∼8%) for patients in the highest CVD risk quartile.

In trials, relative treatment effect modification is commonly assessed using subgroup analyses. Subgroup analyses in REDUCE-IT have previously shown that the relative treatment effect of icosapent ethyl is consistent across subgroups stratified by renal function, smoking status, and history of coronary revascularization, MI, heart failure, and atrial fibrillation.^18–24^ This study, which is the first to assess the efficacy of icosapent ethyl in patients with ASCVD, adds that the relative treatment of icosapent ethyl is also consistent in patients with ASCVD and that its absolute treatment effects in this group are comparable to those in patients with renal dysfunction [estimated glomerular filtration rate (eGFR) <60 mL/min/1.73 m^2^], prior coronary artery bypass graft (CABG), and prior MI.^18,20,22^ However, conventional subgroup analyses have several limitations.^11,13,14,25,26^ First, assessing effect modification in a large number of subgroups leads to a high risk of chance findings.^11,13,14,25,26^ If there is no actual interaction effect, the probability of finding a false-positive treatment interaction is still 5% per tested characteristic (if the most common significance level of 0.05 is applied). Also, true interactions may not be discovered (i.e. yielding false-negative findings), as most trials are not adequately powered to detect subgroup differences.^11,13,14,25,26^ Furthermore, subgroup analyses induce a reference class problem.^11,13,14^ If, for example the relative treatment effect varies across both subgroups of age and subgroups of sex, then it is unclear what the correct effect size is for a young man or a middle-aged woman. Selecting subgroups based on more than one characteristic would lead to a lower number of participants and endpoints per subgroup, further reducing statistical power. To overcome these limitations, in the present study, relative treatment effect heterogeneity was assessed by evaluating the interaction between the treatment and individual baseline risk as predicted by a multivariable risk model, in accordance with previously proposed methods.^11,13,14^ This approach has several advantages over conventional subgroup analyses. First, as this method does not require stratification into large numbers of subgroups, sufficient power may be maintained to detect heterogeneity of treatment effects, while the risk of chance findings is reduced.^11,13,14^ Second, it takes into account multiple characteristics at the same time.^11,13,14^ It is likely that a combination of patient characteristics rather than a single characteristic influences the treatment effect. Also, patient characteristics that are not evaluated in subgroup analyses may contribute to treatment effect heterogeneity as well. As many characteristics are included as, or correlated with predictors in the risk model, heterogeneity based on predicted baseline risk accounts for effect modification by all of these factors. By using a publicly available risk model, i.e. the SMART2 and SMART-REACH risk models in this study, the results can be directly applied and used for individualized clinical decision-making in clinical practice.

Applying this method to REDUCE-IT revealed no significant interaction between baseline risk and the relative treatment effect of icosapent ethyl. Numerically, the relative treatment effect was larger in patients with a lower baseline risk, but as the treatment-by-risk interaction was non-significant, this finding should be interpreted with caution. If one were to speculate on possible reasons for the more favourable relative treatment effect observed at the lower end of the risk spectrum, one could think of several explanations. First, icosapent ethyl and other interventions targeting CVD risk factors may be more effective in the earlier stages of atherosclerotic disease, as in this stage the development of clinically significant plaques may still be avoided. While in patients with more advanced disease, lowering triglycerides or levels of other risk factors may halt the progression of plaques, but may not cause reversal to a state in which there is no or only minimal atherosclerosis. This is supported by studies of the relation between LDL-c and CVD events, showing that the risk of CVD largely depends on the cumulative exposure to LDL-c at a younger age and that a genetically determined lower LDL-c (from birth) has a greater influence on CVD risk than the same magnitude of LDL-c reduction with a statin later in life.^27–29^ Also, in trials, statins have led to larger relative reductions in the risk of CVD per 1 mmol/L LDL-c reduction in primary as compared with secondary prevention and younger as compared with older individuals.^30,31^ Second, in low-risk patients from REDUCE-IT, their elevated triglyceride level may be (one of) the main driver(s) of their CVD risk, and lowering triglycerides may therefore have a relatively large impact on their risk of future events. Whereas high-risk patients mostly have multiple other and more dominant risk factors (e.g. hypertension, diabetes mellitus, smoking) not modified by icosapent ethyl that, even if triglyceride levels are reduced, may still trigger CVD events. In other words, it may be more difficult to prevent events in high-risk patients with multiple risk factors, potentially explaining the smaller relative risk reduction observed in these patients.

Even if the relative treatment effect of icosapent ethyl is equal for all patients, there are still substantial differences in the absolute treatment effect due to variation in the absolute baseline CVD risk. If the overall trial HR (0.72 for 3-point MACE) would apply to all patients, the expected 5-year absolute reduction in 3-point MACE would range from 2.4% in the lowest risk quartile (baseline 5-year risk 9.0%) to 8.1% in the highest risk quartile (baseline 5-year risk 33.7%). The fact that, in cases of an equal relative treatment effect, ARRs are greater and so NNTs are smaller in higher-risk patients means that interventions are usually more (cost-)effective in this group. This is one of the main reasons why in guidelines treatments such as icosapent ethyl are often recommended specifically for patients with a high residual risk. However, the current study showed that in REDUCE-IT, the absolute treatment effects of icosapent ethyl in lower- vs. higher-risk patients are in fact much closer together, with an ARR and 5-year CIR that ranged from 3.9% and 4.8% in the lowest to 5.6% and 7.7% in the highest CVD risk quartile. This indicates that icosapent ethyl also leads to substantial absolute reductions in CVD risk in ASCVD patients with elevated triglycerides and a relatively low residual risk. In addition, as lower-risk patients generally have a longer remaining life expectancy and can therefore be treated over a longer period of time, their expected lifetime benefits often exceed those of high-risk patients, as was also shown in this study. Had total events (i.e. also including second, third, and fourth or more events) been considered, the benefits of icosapent ethyl may have been even larger for both low- and high-risk patients.^32^ These results may support a broader use of icosapent ethyl than currently recommended by the ESC and AHA/ACC guidelines, and expert consensus documents.^6–10^

### Study limitations

The median follow-up duration in the trial was 4.8 years and the analyses of observed treatment effects were therefore limited to 5 years. In practice, icosapent ethyl will mostly be continued lifelong. Predicted lifetime benefits were presented in this study, but could only be validated up to 5 years. Like any trial, REDUCE-IT had eligibility criteria, so the results may not be generalizable to all patients in clinical practice. Also, in routine practice, greater non-adherence may be expected, resulting in smaller treatment effects. Side-effects and potential heterogeneity of these side effects according to baseline risk were not evaluated. But in REDUCE-IT, the rates of adverse events did not differ significantly between the icosapent ethyl and placebo group, with the exception of atrial fibrillation (5.3% vs. 3.9%) and peripheral oedema (6.5% vs. 5.0%).^5^ Treatment effect heterogeneity based on effect modifiers that do not influence CVD risk and are not associated with factors that do, is not detected with the methods applied in this study. In practice, greater heterogeneity may be present. The numbers of patients and events in this study allowed for the assessment of treatment effects in quartiles of baseline risk. In the case of a larger sample size, treatment effects could have been assessed in a larger number of risk groups, and the continuous relation between baseline risk and treatment effects could have been more accurately estimated.

## Conclusions

Among patients with ASCVD and elevated triglyceride levels, icosapent ethyl significantly reduces the risk of MACE across all quartiles of baseline CVD risk. The absolute treatment effects increase with increasing baseline CVD risk, but are already substantial for patients in the lowest risk quartile.

## Supplementary Material

pvae030_Supplemental_File

## Notes

**Conflict of interest:** Dr Bhatt served as the Principal Investigator of REDUCE-IT with research funding from Amarin to his institutions and discloses the following relationships—Advisory Board: Angiowave, Bayer, Boehringer Ingelheim, CellProthera, Cereno Scientific, Elsevier Practice Update Cardiology, High Enroll, Janssen, Level Ex, McKinsey, Medscape Cardiology, Merck, MyoKardia, NirvaMed, Novo Nordisk, PhaseBio, PLx Pharma, Stasys; Board of Directors: American Heart Association New York City, Angiowave (stock options), Bristol Myers Squibb (stock), DRS.LINQ (stock options), High Enroll (stock); Consultant: Broadview Ventures, GlaxoSmithKline, Hims, SFJ, Youngene; Data Monitoring Committees: Acesion Pharma, Assistance Publique-Hôpitaux de Paris, Baim Institute for Clinical Research (formerly Harvard Clinical Research Institute, for the PORTICO trial, funded by St. Jude Medical, now Abbott), Boston Scientific (Chair, PEITHO trial), Cleveland Clinic, Contego Medical (Chair, PERFORMANCE 2), Duke Clinical Research Institute, Mayo Clinic, Mount Sinai School of Medicine (for the ENVISAGE trial, funded by Daiichi Sankyo; for the ABILITY-DM trial, funded by Concept Medical; for ALLAY-HF, funded by Alleviant Medical), Novartis, Population Health Research Institute; Rutgers University (for the NIH-funded MINT Trial); Honoraria: American College of Cardiology (Senior Associate Editor, Clinical Trials and News, ACC.org; Chair, ACC Accreditation Oversight Committee), Arnold and Porter law firm (work related to Sanofi/Bristol-Myers Squibb clopidogrel litigation), Baim Institute for Clinical Research (formerly Harvard Clinical Research Institute; RE-DUAL PCI clinical trial steering committee funded by Boehringer Ingelheim; AEGIS-II executive committee funded by CSL Behring), Belvoir Publications (Editor in Chief, Harvard Heart Letter), Canadian Medical and Surgical Knowledge Translation Research Group (clinical trial steering committees), CSL Behring (AHA lecture), Cowen and Company, Duke Clinical Research Institute (clinical trial steering committees, including for the PRONOUNCE trial, funded by Ferring Pharmaceuticals), HMP Global (Editor in Chief, Journal of Invasive Cardiology), Journal of the American College of Cardiology (Guest Editor; Associate Editor), K2P (Co-Chair, interdisciplinary curriculum), Level Ex, Medtelligence/ReachMD (CME steering committees), MJH Life Sciences, Oakstone CME (Course Director, Comprehensive Review of Interventional Cardiology), Piper Sandler, Population Health Research Institute (for the COMPASS operations committee, publications committee, steering committee, and USA national co-leader, funded by Bayer), WebMD (CME steering committees), Wiley (steering committee); Other: Clinical Cardiology (Deputy Editor); Patent: Sotagliflozin (named on a patent for sotagliflozin assigned to Brigham and Women's Hospital who assigned to Lexicon; neither I nor Brigham and Women's Hospital receive any income from this patent); Research Funding: Abbott, Acesion Pharma, Afimmune, Aker Biomarine, Alnylam, Amarin, Amgen, AstraZeneca, Bayer, Beren, Boehringer Ingelheim, Boston Scientific, Bristol-Myers Squibb, Cardax, CellProthera, Cereno Scientific, Chiesi, CinCor, Cleerly, CSL Behring, Eisai, Ethicon, Faraday Pharmaceuticals, Ferring Pharmaceuticals, Forest Laboratories, Fractyl, Garmin, HLS Therapeutics, Idorsia, Ironwood, Ischemix, Janssen, Javelin, Lexicon, Lilly, Medtronic, Merck, Moderna, MyoKardia, NirvaMed, Novartis, Novo Nordisk, Otsuka, Owkin, Pfizer, PhaseBio, PLx Pharma, Recardio, Regeneron, Reid Hoffman Foundation, Roche, sanofi, Stasys, Synaptic, The Medicines Company, Youngene, 89Bio; Royalties: Elsevier (Editor, Braunwald's Heart Disease); Site Co-Investigator: Abbott, Biotronik, Boston Scientific, CSI, Endotronix, St. Jude Medical (now Abbott), Philips, SpectraWAVE, Svelte, Vascular Solutions; Trustee: American College of Cardiology; Unfunded Research: FlowCo. Dr Steg discloses the following relationships: Research grants: Amarin, Bayer, sanofi, Servier; Clinical Trials (Steering committee, CEC, DSMB): Amarin, AstraZeneca, Bayer, Boehringer Ingelheim, Bristol-Myers Squibb, Idorsia, Janssen, Novartis, Pfizer, sanofi, Servier; Consulting or speaking: Amarin, Amgen, BMS/Myokardia, Novartis, Novo-Nordisk, Regeneron; Journals: Senior Associate Editor at Circulation. The other authors declared no conflicts of interest.

## Data availability

The data underlying this article were provided by Amarin Pharma. Data will be shared on reasonable request to the corresponding author with permission of the principal investigator.
