# Supplementary material for: Effects of icosapent ethyl according to baseline residual risk in patients with atherosclerotic cardiovascular disease: results from REDUCE-IT
Source: Eur Heart J Cardiovasc Pharmacother. 2024 Apr 27;10(6):488–99. doi: 10.1093/ehjcvp/pvae030 (PMC11873788; doi:10.1093/ehjcvp/pvae030)
Supplement: pvae030_Supplemental_File [file pvae030_supplemental_file.docx]

**Supplemental Material**

**Table of contents (*use Ctrl + click to navigate*)**

[**Supplemental Methods**](#supmeth1)

[Methods S1. Recalibration of the SMART2 and SMART-REACH risk models](#supmeth1)

[Methods S2. Estimation of the lifetime benefits of icosapent ethyl](#supmeth2)

[Methods S3. Continuous analyses of the relation between baseline risk and the treatment effects of icosapent ethyl](#supmeth3)

[**Supplemental Tables**](#suptab1b)

[Table S1. Eligibility criteria of REDUCE-IT](#suptab1b)

[Table S2. Predictors in the SMART2 and SMART-REACH risk models](#suptab2)

[Table S3. Outcome definitions](#suptab3)

[Table S4. Missing data](#suptab4)

[**Supplemental Figures**](#supfig1)

[Figure S1. Efficacy of icosapent ethyl in patients with ASCVD](#supfig1)

[Figure S2. Calibration for 5-point MACE across CVD risk quartiles](#supfig2b)

[Figure S3. Validation of the SMART2 and SMART-REACH models in octiles of risk](#supfig3)

[Figure S4. Lifetime benefit for 5-point MACE across CVD risk quartiles](#supfig4)

[Figure S5. Continuous relation between baseline risk and the effects of icosapent ethyl on 5-point MACE](#supfig5)

[**Supplemental References**](#supref)

**Methods S1. Recalibration of the SMART2 and SMART-REACH risk models**

First, the SMART2 risk score was applied to all patients in the ASCVD study population, predicting each patient’s 5-year risk of 3-point MACE, using the coefficients and 5-year baseline hazard published in the original report.^1^ To take into account the effect of icosapent ethyl on the risk of 3-point MACE, allocation to icosapent ethyl was added to the model as a dummy variable using the hazard ratio from the trial (HR = 0.72 for participants in the secondary prevention cohort).^2^ Next, the expected vs observed (E/O) ratio was calculated by dividing the mean predicted 5-year risk of 3-point MACE (as predicted by the SMART2 risk score including allocation to icosapent ethyl) by the mean observed 5-year risk of 3-point MACE in the study population. To recalibrate the model to the underlying event rate in the study population, the logarithm of the E/O ratio was subtracted from the linear predictor of the model. In this way, the baseline hazard is recalibrated and tailored to the study population, while keeping the original model coefficients. When the recalibrated model was then used to predict each patient’s baseline 5-year risk of 3-point MACE, i.e. a patient’s predicted risk without icosapent ethyl, the dummy variable for icosapent ethyl was removed from the model.

This methodology was also used to recalibrate the SMART-REACH model. Besides a function for 3-point MACE, the SMART-REACH model also consists of a separate function for non-cardiovascular mortality. Recalibration was also performed for this outcome, using a separate E/O ratio. Like the E/O ratio for 3-point MACE was subtracted from the linear predictor of the function for 3-point MACE, the E/O ratio for non-cardiovascular mortality was subtracted from the linear predictor of the function for non-cardiovascular mortality.

The same methodology was used to make the models suitable for the prediction of 5-point MACE, instead of 3-point MACE (the outcome for which the models were originally developed). For this, the original model coefficients were used, i.e. the assumption was made that the association between the predictors and 5-point MACE was equal to the association between the predictors and 3-point MACE. For both models, the baseline hazard was recalibrated to match the underlying event rate for 5-point MACE in the study population, by calculating the E/O ratio for this outcome and subtracting the E/O ratio from the linear predictor of the model.

**Methods S2. Estimation of the lifetime benefits of icosapent ethyl**

Lifetime risk of MACE and the lifetime benefits of icosapent ethyl were predicted with the SMART-REACH model, in accordance with previously developed methods.^3,4^ First, the SMART-REACH model (recalibrated to the study population; see Methods S1) was used to predict the MACE-free survival without icosapent ethyl for all individuals in the ASCVD study population. This was done by making use of life-tables. Starting from the age of each patient at baseline, the risk of MACE (a_t_) and the risk of non-cardiovascular mortality (b_t_) were estimated for each consecutive life-year, up to the maximum age of 90 years. A MACE-free survival probability (p_t_) was obtained for each life-year, by subtracting MACE risk and non-cardiovascular mortality risk from 1 (p_t_ = 1 – a_t_ – b_t_). The probability of being alive and free of MACE at the start of each life-year (e_t_), was calculated by multiplying the MACE-free survival probabilities of all the previous life-years (e.g. for a 60-year old: e_t=90_ = p_t=60_ * p_t=61_ * p_t=62_ * ……. * p_t=87_ * p_t=88_ * p_t=89_). Altogether, these predictions form an individual life-table for each patient. For each risk quartile, the average MACE-free survival curve was drawn by taking the mean of the MACE-free survival probabilities (derived from the individual life-tables) of the patients within the risk quartile, at 1 to 30 years after the starting age. The median MACE-free survival without icosapent ethyl in each risk quartile was calculated as the time where the MACE-free survival curve crossed 50%.

Next, the SMART-REACH model was combined with the relative treatment effect of icosapent ethyl for patients with ASCVD derived from the original trial report (HR = 0.72 for 3-point MACE, and HR = 0.73 for 5-point MACE).^2^ The model combined with the relative treatment effect was then used to predict the MACE-free survival with icosapent ethyl for all patients. Again, the mean MACE-free survival probability for all patients within a risk quartile at 1-year time intervals was used to draw the average MACE-free survival curve for each risk quartile, and the median MACE-free survival was calculated as the time where the curve crossed 50%. Within each risk quartile, the lifetime benefit from icosapent ethyl was defined as the difference between the median MACE-free survival with and without icosapent ethyl, and was expressed as life-years without MACE gained.

**Methods S3. Continuous analyses of the relation between baseline risk and the treatment effects of icosapent ethyl**

The continuous relation between the predicted baseline 5-year risk of MACE and the relative treatment effect of icosapent ethyl was assessed by deriving a Cox proportional hazards model including the following terms: allocation to icosapent ethyl, predicted baseline 5-year risk, and the interaction between icosapent ethyl and baseline risk as a restricted cubic spline (with four knots). This model was used to estimate the course of the relative treatment effect across the spectrum of baseline risk. Corresponding 95% confidence intervals were derived by repeating this process in 10,000 bootstrap samples. The 2.5^th^ and 97.5^th^ percentile of the bootstrap samples were used as the lower and upper limit respectively.

Measures of absolute treatment effects such as the absolute risk reduction (ARR) and 5-year cumulative incidence reduction (CIR) cannot be derived directly from a statistical model. So, instead these were determined in increasingly small risk groups. The ARR was defined as the proportion of patients with an event in the placebo group minus the proportion of patients with an event in the icosapent ethyl group at the end of follow-up. The standard error (SE) of the ARR was calculated using the following formula: $\sqrt{\text{(E\%}\text{Placebo }\text{* (1 - E\%}\text{Placebo}\text{) / N}\text{Placebo}\text{)}+(\text{E\%}\text{Icosapent ethyl }\text{* (1 - E\%}\text{Icosapent ethyl}\text{) / N}\text{Icosapent ethyl}\text{)}}$with *E%* being the proportion of patients with an event in each group, and *N* being the total number of patients in each group. First, the ARR and corresponding SE were determined in the total population. Then, this was done in two risk groups, divided at the median of the baseline 5-year risk of MACE. Subsequently, ARRs and corresponding SEs were also determined in tertiles, quartiles, and quintiles of baseline risk. For each risk group, the mean baseline 5-year risk of MACE was also calculated. This yielded fifteen ARR estimates, associated with varying SEs (the smaller the risk group in which the ARR was determined, the larger the SE), and varying levels of baseline risk (e.g. the ARR determined in the lowest risk tertile belonged to a lower baseline risk than the ARR determined in the highest risk quartile). The continuous relation between the ARR and baseline risk was assessed using a linear model regressing the ARR on baseline risk, with baseline risk as a restricted cubic spline. The model was weighted for the accuracy of each ARR estimate, i.e. the inverse of its SE (1/SE). This model was used to estimate the course of the ARR across the spectrum of baseline risk. This process was repeated in 10,000 bootstrap samples. The mean of the bootstrap samples was used as the final estimate for the course of the ARR over baseline risk. The 2.5^th^ and 97.5^th^ percentiles were used as the lower and upper limits of the 95% confidence intervals respectively.

The 5-year CIR was calculated as the difference between the Kaplan-Meier estimate of the cumulative incidence of MACE in the placebo as compared to the icosapent ethyl group at 5 years follow-up. The SE of the 5-year CIR was calculated using the SEs of the two individual Kaplan-Meier estimates of the cumulative incidence of MACE at 5 years (one for the placebo group and one for the icosapent ethyl group), based on the following formula: $\sqrt{(SE\text{Placebo}\text{)}\text{2 }+(SE\text{Icosapent ethyl}\text{)}\text{2}}$. The course of the 5-year CIR across the spectrum of baseline risk was determined by calculating the 5-year CIR in increasingly small risk groups and repeating this process in 10,000 bootstrap samples, using the same methods as for the ARR (see above).

For the continuous relation between baseline risk and the lifetime benefit of icosapent ethyl, first, the lifetime benefit in terms of life-years without MACE gained was estimated for each individual in the study population using the SMART-REACH model combined with the overall relative treatment effect of icosapent ethyl (see Methods S2). Then, a linear model was derived regressing lifetime benefit on baseline risk, with baseline risk as a restricted cubic spline. This model was used to estimate the course of the lifetime benefit over baseline risk. Again, 95% confidence intervals were derived from 10,000 bootstrap samples.

| **Table S1. Eligibility criteria of REDUCE-IT** |  |
| --- | --- |
| **Inclusion criteria**^2,5^ | |
| Age ≥45 years (if secondary prevention; see below) or ≥50 years (if primary prevention; see below) | |
| Secondary prevention (one of the following)^a^:   - Documented CAD:   - Multi-vessel CAD (≥50% stenosis in at least two major epicardial coronary arteries with or without antecedent revascularization), or;   - Prior MI, or;   - Hospitalization for NSTE-ACS with ST-segment deviation or biomarker positivity. - Documented cerebrovascular or carotid disease:   - Prior ischemic stroke, or;   - Symptomatic carotid arterial stenosis ≥50%, or;   - Asymptomatic carotid arterial stenosis ≥70%, or;   - History of carotid revascularization. - Documented PAD:   - ABI <0.9 with intermittent claudication, or;   - History of aorto-iliac or peripheral arterial intervention.   Primary prevention^a^:   - Diabetes mellitus (type 1 or type 2) requiring treatment with medication, AND; - ≥1 CVD risk factor(s):   - Age ≥55 years (men) or ≥65 years (women)   - Current smoking (or stopped smoking <3 months before first visit)   - Hypertension (≥140/90 mmHg) or on antihypertensive medication   - HDL-c ≤40 mg/dL (≤1.03 mmol/L) for men or ≤50 mg/dL (≤1.29 mmol/L) for women   - Hs-CRP >3.0 mg/L   - Renal dysfunction (CrCL >30 and <60 mL/min)   - Retinopathy   - Micro- or macroalbuminuria   - ABI <0.9 | |
| Fasting triglyceride level ≥135 mg/dL (≥1.52 mmol/L) and <500 mg/dL (<5.65 mmol/L) | |
| LDL-c >40 mg/dL (>1.03 mmol/L) and ≤100 mg/dL (≤2.59 mmol/L) | |
| On stable statin therapy (± ezetimibe) for ≥4 weeks prior to randomization | |
| Agree to follow a physician-recommended diet | |

| **Exclusion criteria**^2,5^ |
| --- |
| Severe heart failure (NYHA class IV) |
| Life-threatening disease with life expectancy <2 years |
| Severe liver disease |
| HbA1c >10.0% (>86 mmol/mol) |
| Poorly controlled hypertension (≥200/100 mmHg) |
| Planned coronary intervention or non-cardiac major surgical procedure |
| Familial lipoprotein lipase deficiency, apolipoprotein C-II deficiency, or familial dysbetalipoproteinemia |
| Intolerance or hypersensitivity to statin therapy |
| Hypersensitivity to fish and/or shellfish, or ingredients of the study product or placebo |
| History of acute or chronic pancreatitis |
| Malabsorption syndrome or chronic diarrhea |
| Use of non-study drug, non-statin lipid-altering medications, supplements, or foods including:   - Niacin >200 mg/d - Fibrates - OM-3 fatty acid medications - Supplements containing OM-3 fatty acids - Bile acid sequestrants - PCSK9 inhibitors |
| Use of one of the following medications:   - Tamoxifen, estrogens, progestins, thyroid hormone therapy, systemic corticosteroids, cyclophosphamide, or systemic retinoids |
| Known AIDS |
| Requirement for dialysis or CrCl <30 mL/min |
| CK concentration >5 × ULN or CK elevation due to muscle disease |
| Pregnant or breastfeeding women, or women of child-bearing potential not using an acceptable form of birth control |
| ^a^ For the current study, only participants who met the criteria for the secondary prevention stratum in REDUCE-IT were included.  Abbreviations: ABI = ankle-brachial index, AIDS = acquired immunodeficiency syndrome, CAD = coronary artery disease, CK = creatine kinase, CrCl = creatinine clearance, CVD = cardiovascular disease, HbA1c = hemoglobin A1c, HDL-c = high-density lipoprotein cholesterol, hs-CRP = high-sensitivity C-reactive protein, LDL-c = low-density lipoprotein cholesterol, MI = myocardial infarction, NSTE-ACS = non-ST-segment elevation acute coronary syndrome, NYHA = New York Heart Association, OM-3 = omega-3, PAD = peripheral artery disease, PCSK9 = proprotein convertase subtilisin/kexin type 9, ULN = upper limit of normal. |

| **Table S2. Predictors in the SMART2 and SMART-REACH risk models** | |
| --- | --- |
| **Model** | **Predictors** |
| SMART2 risk score^1^ | Age |
|  | Sex |
|  | Current smoking |
|  | History of coronary artery disease |
|  | History of cerebrovascular disease |
|  | History of peripheral artery disease |
|  | History of abdominal aortic aneurysm |
|  | Years since first ASCVD diagnosis |
|  | Diabetes mellitus |
|  | Systolic blood pressure |
|  | Non-HDL-cholesterol |
|  | Estimated glomerular filtration rate |
|  | High-sensitivity CRP |
| SMART-REACH model^4^ | Age |
|  | Sex |
|  | Current smoking |
|  | Number of ASCVD locations^a^ |
|  | Diabetes mellitus |
|  | History of atrial fibrillation |
|  | History of heart failure |
|  | Systolic blood pressure |
|  | Total cholesterol |
|  | Creatinine |
| ^a^ Number of ASCVD locations out of coronary artery disease, cerebrovascular disease, and peripheral artery disease (one, two, or three).  Abbreviations: ASCVD = atherosclerotic cardiovascular disease, CRP = C-reactive protein, HDL = high-density lipoprotein. | |

| **Table S3. Outcome definitions** | |
| --- | --- |
| **Outcome** | **Definition**^2,6^ |
| Myocardial infarction | Evidence of myocardial necrosis (≥1 cardiac biomarker(s) >URL, or post-mortem pathological evidence of acute MI), combined with a clinical presentation consistent with myocardial ischemia, electrocardiographic changes (ST elevation or depression, T-wave inversion, or pathological Q-waves), or evidence from myocardial or coronary artery imaging (loss of viable myocardium, regional wall motion abnormality, or thrombosis/occlusion of coronary artery), including silent MI (new pathological Q-waves, imaging evidence of loss of viable myocardium, or autopsy evidence of a healed or healing MI, without evidence of acute MI). |
| Stroke | Acute episode of focal cerebral, spinal, or retinal dysfunction caused by an infarction of central nervous system tissue, or a nontraumatic intraparenchymal, intraventricular, or subarachnoid hemorrhage. |
| Cardiovascular death | Death resulting from myocardial infarction, heart failure, or stroke, sudden cardiac death, or death due to other cardiovascular causes (e.g. pulmonary embolism, aortic aneurysm rupture, peripheral artery disease, complications of cardiac surgery or revascularization). |
| Coronary revascularization | A catheter-based or open surgical procedure designed to improve myocardial blood flow, i.e. PCI or CABG. |
| Unstable angina | Ischemic discomfort (angina or equivalent symptoms) ≥10 minutes in duration occurring at rest or in an accelerating pattern with frequent episodes associated with progressively decreased exercise capacity, prompting an unscheduled hospitalization within 24 hours of the most recent symptoms, combined with electrocardiographic changes (ST elevation/depression, or T-wave inversion), a positive exercise stress test, evidence from myocardial or coronary artery imaging (wall motion abnormality, perfusion defect/deficit, or lesion/thrombus in coronary artery), or the need for coronary revascularization, with negative cardiac biomarkers and no evidence of acute MI. |
| Outcomes were defined in accordance with the Standardized Definitions for Cardiovascular and Stroke Endpoint Events in Clinical Trials.^6^ All events were adjudicated by an independent clinical endpoint committee blinded for the trial-group assignment.^2^  Abbreviations: CABG = coronary artery bypass graft, MI = myocardial infarction, PCI = percutaneous coronary intervention, URL = upper reference limit. | |

| **Table S4. Missing data** | |
| --- | --- |
| **Variable** | **Missing values, n (%)** |
| Age | 0 (0.0%) |
| Sex | 0 (0.0%) |
| Current smoking | 2 (0.0%) |
| Number of ASCVD locations | 0 (0.0%) |
| History of coronary artery disease | 0 (0.0%) |
| History of cerebrovascular disease | 0 (0.0%) |
| History of peripheral artery disease | 0 (0.0%) |
| History of abdominal aortic aneurysm | 0 (0.0%) |
| Years since first ASCVD diagnosis | 570 (9.9%) |
| Diabetes mellitus | 3 (0.1%) |
| History of atrial fibrillation | 0 (0.0%) |
| History of heart failure | 3 (0.1%) |
| Systolic blood pressure | 10 (0.2%) |
| Total cholesterol | 5 (0.1%) |
| Non-HDL-cholesterol | 17 (0.3%) |
| Creatinine | 6 (0.1%) |
| Estimated glomerular filtration rate | 6 (0.1%) |
| High-sensitivity CRP | 4 (0.1%) |
| Overview of the missing data for predictor variables from the SMART2 and SMART-REACH risk models in the study population (n = 5,785).  Abbreviations: ASCVD = atherosclerotic cardiovascular disease, CRP = C-reactive protein, HDL = high-density lipoprotein. | |

**Figure S1. Efficacy of icosapent ethyl in patients with ASCVD**


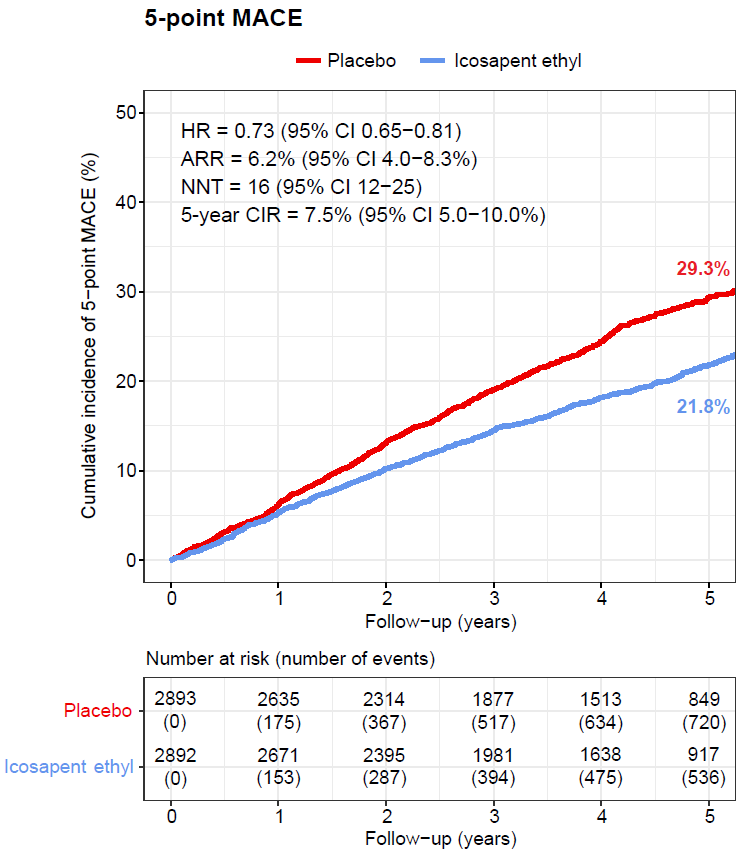
**
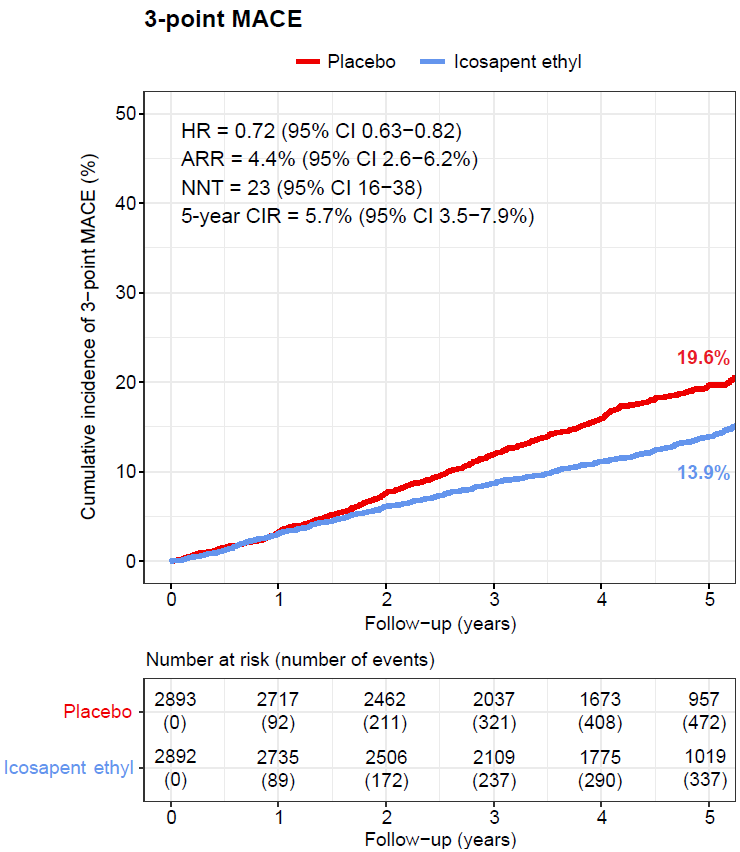
**

**B**

**A**

**C**


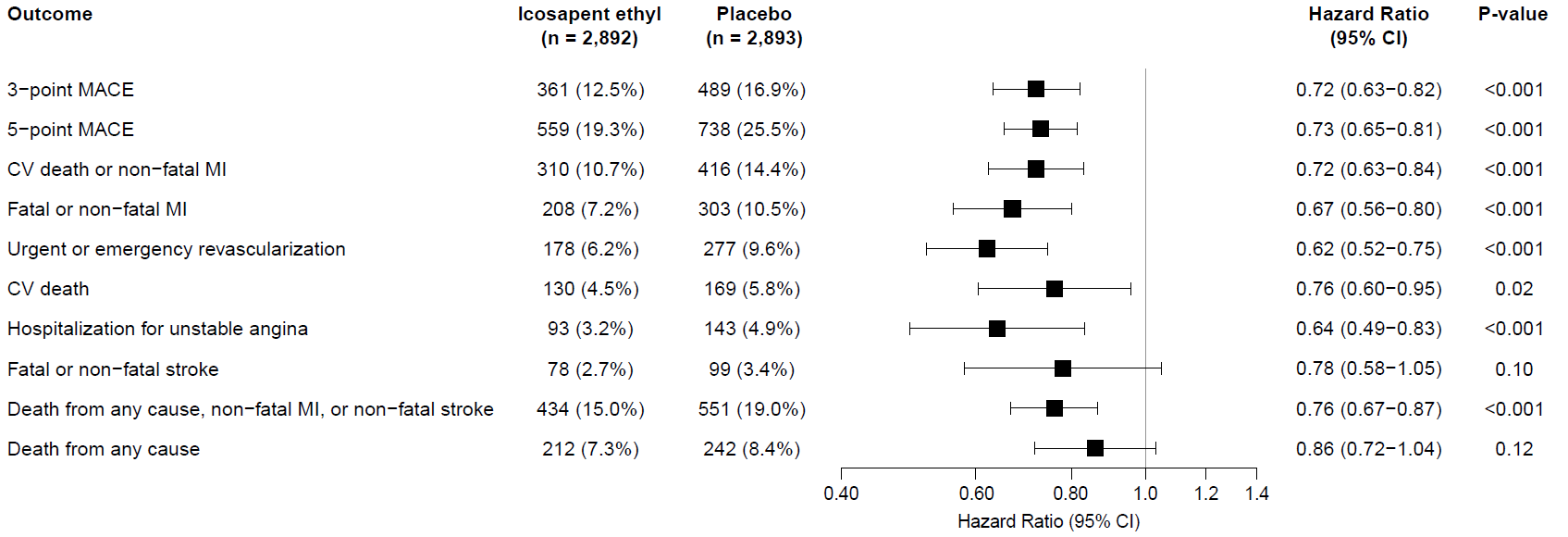


Kaplan-Meier curves of the cumulative incidence of 3-point MACE (A) and 5-point MACE (B), and hazard ratios for all outcomes (C) in REDUCE-IT participants with ASCVD (n = 5,785). ARRs were calculated as the proportion of patients with an event in the placebo group minus the proportion of patients with an event in the icosapent ethyl group at the end of follow-up. The red and blue numbers indicate the cumulative incidence at 5 years follow-up in the placebo and icosapent ethyl group respectively. The 5-year cumulative incidence reductions (CIRs) were calculated as the difference between the red and blue numbers. Three-point MACE is a composite of non-fatal myocardial infarction, non-fatal stroke, or cardiovascular death. Five-point MACE additionally includes coronary revascularization, and unstable angina.

Abbreviations: ARR = absolute risk reduction, CI = confidence interval, CIR = cumulative incidence reduction, CVD = cardiovascular disease, HR = hazard ratio, MACE = major adverse cardiovascular events, NNT = number needed to treat.

**Figure S2. Calibration for 5-point MACE across CVD risk quartiles**


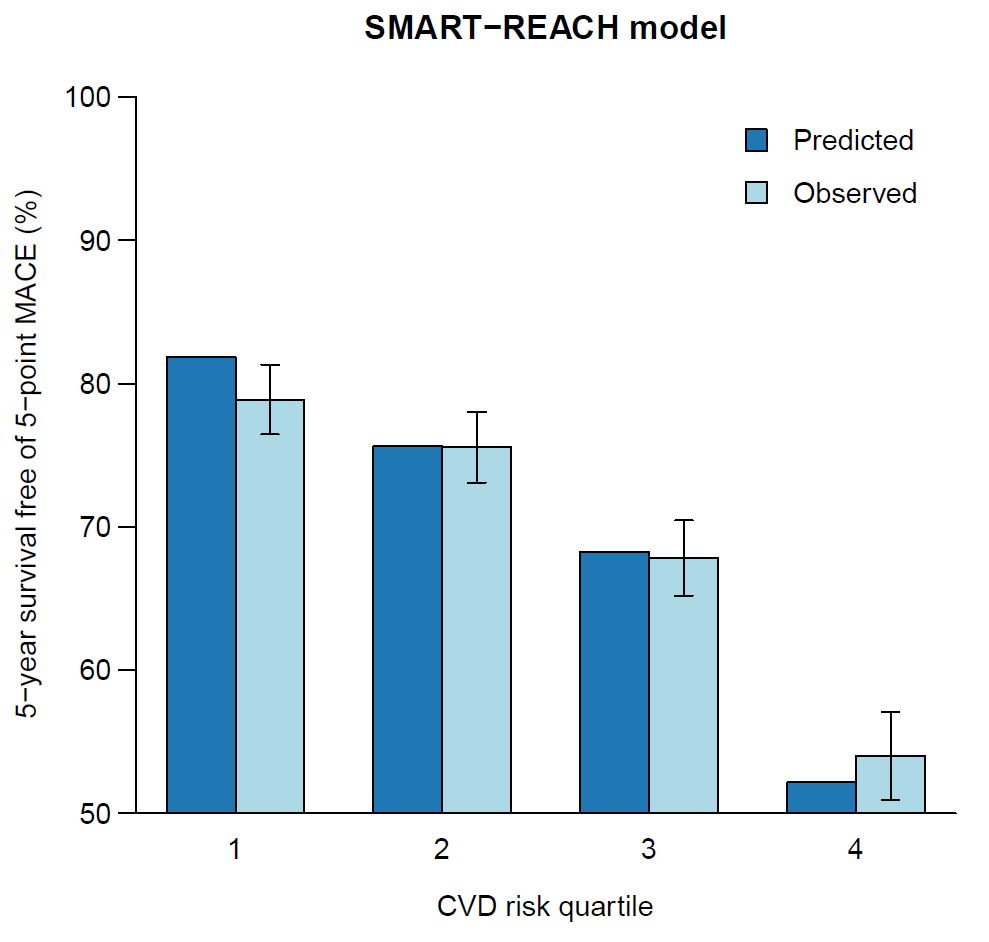
**
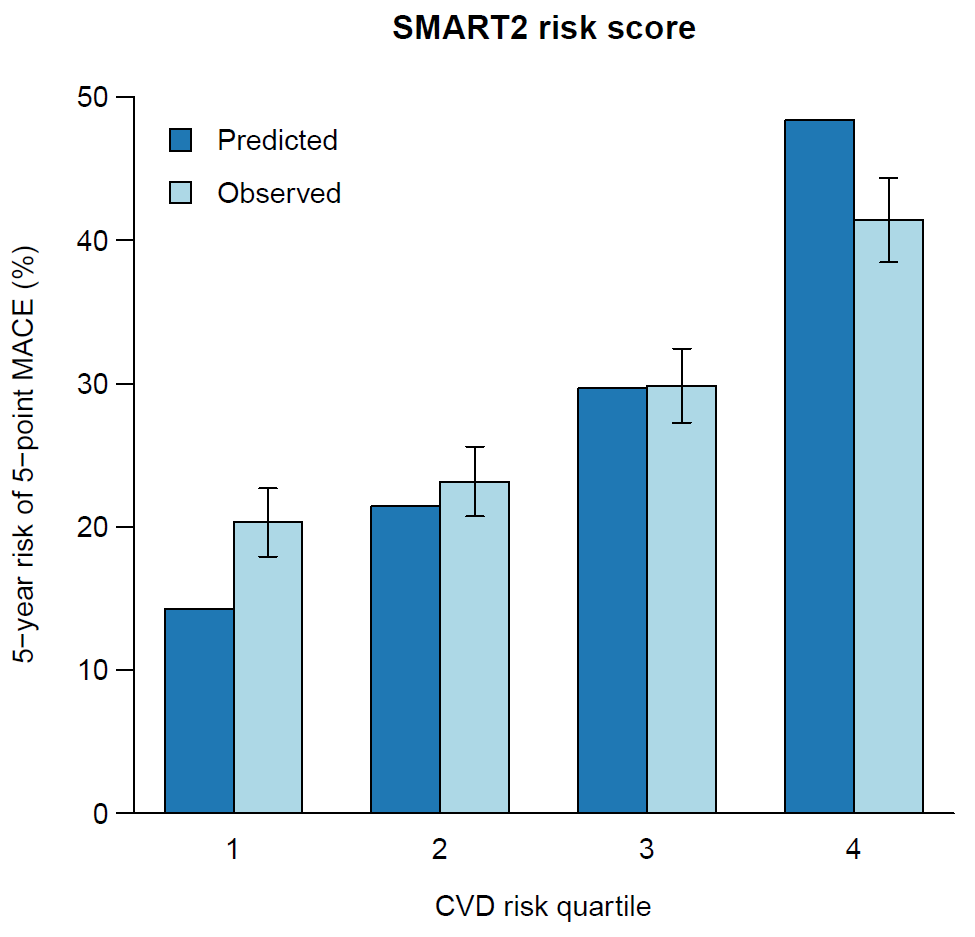
**

**B**

**A**

Mean predicted 5-year risk (by the recalibrated SMART2 risk score) vs observed 5-year risk of 5-point MACE (A), and mean predicted 5-year survival (by the recalibrated SMART-REACH model) vs observed 5-year survival free of 5-point MACE (B) across the CVD risk quartiles. Error bars represent 95% confidence intervals. Five-point MACE is a composite of non-fatal myocardial infarction, non-fatal stroke, cardiovascular death, coronary revascularization, or unstable angina.

Abbreviations: CVD = cardiovascular disease, MACE = major adverse cardiovascular events.

**Figure S3. Validation of the SMART2 and SMART-REACH models in octiles of risk**

3-point MACE

**
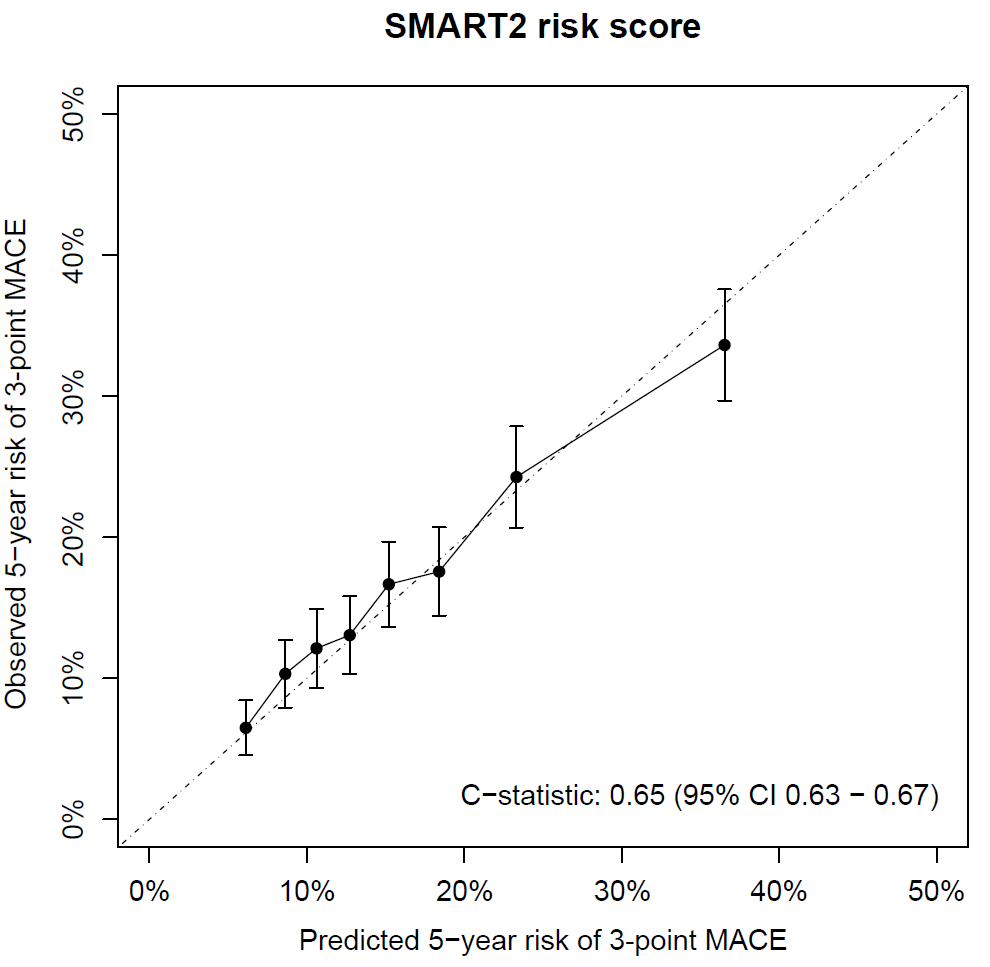

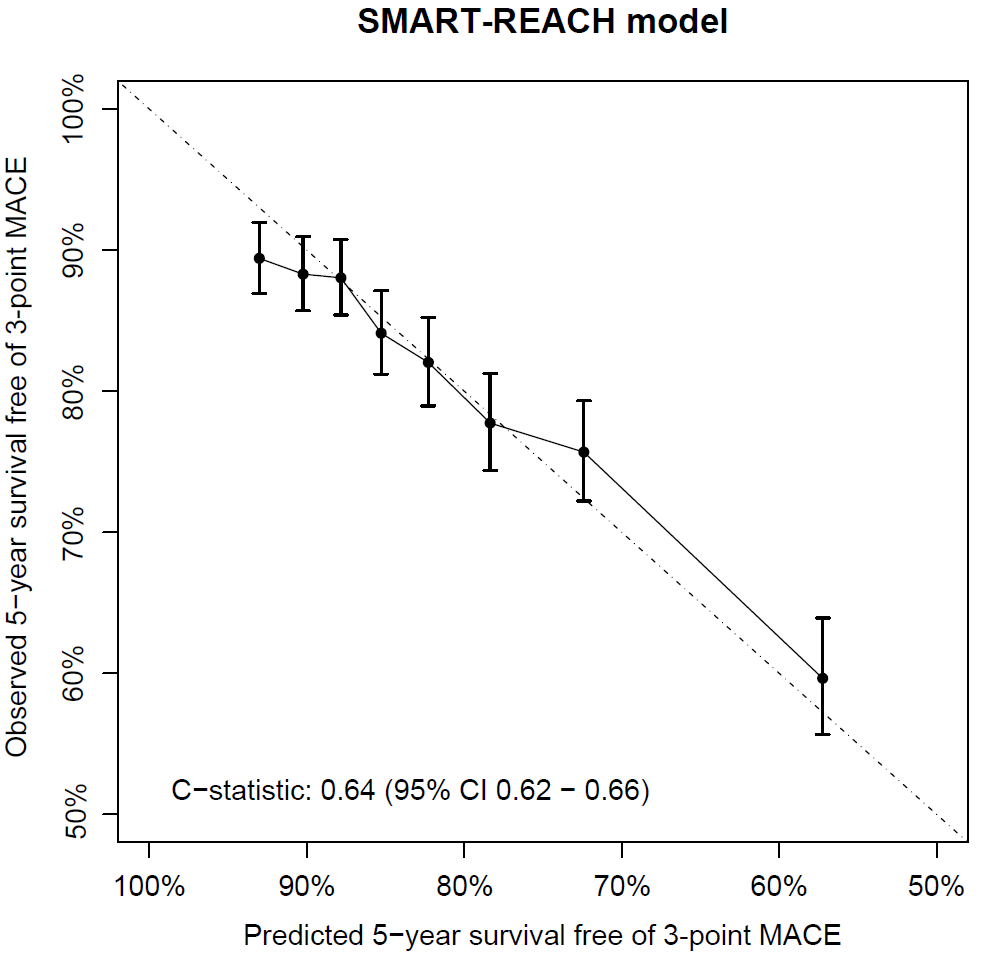
**


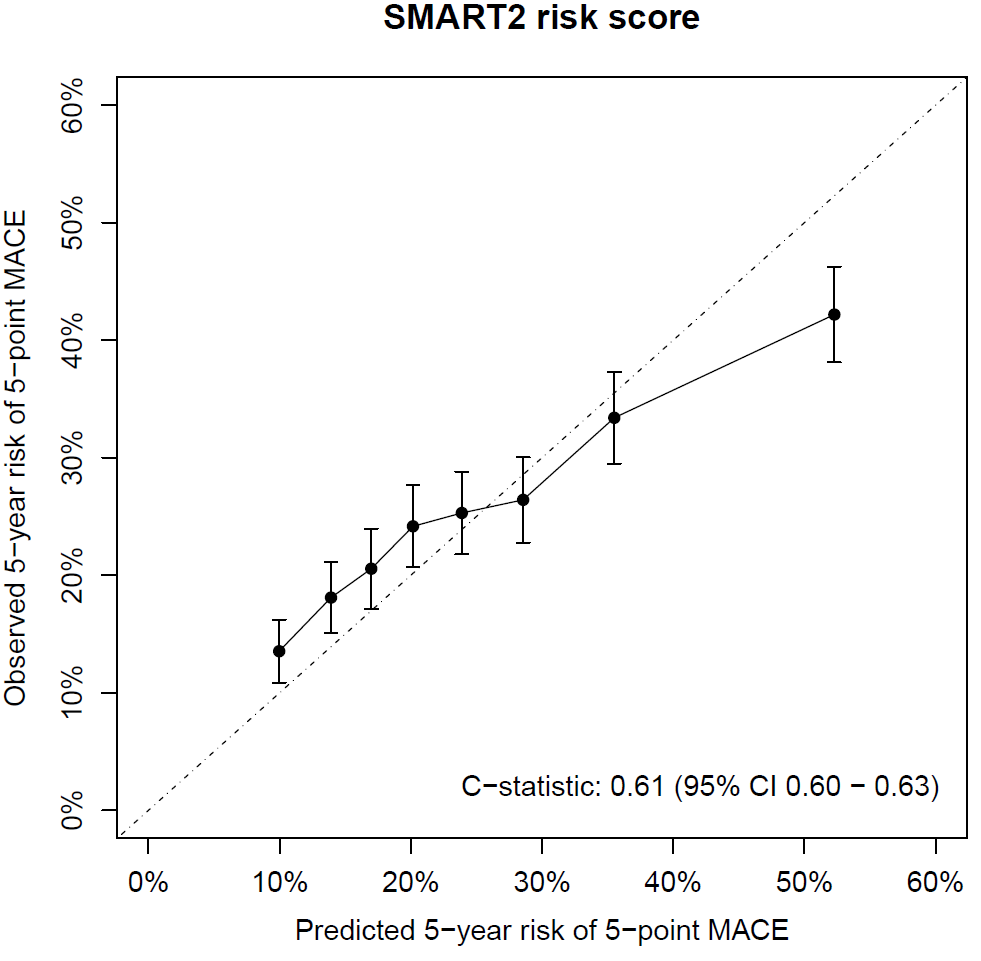

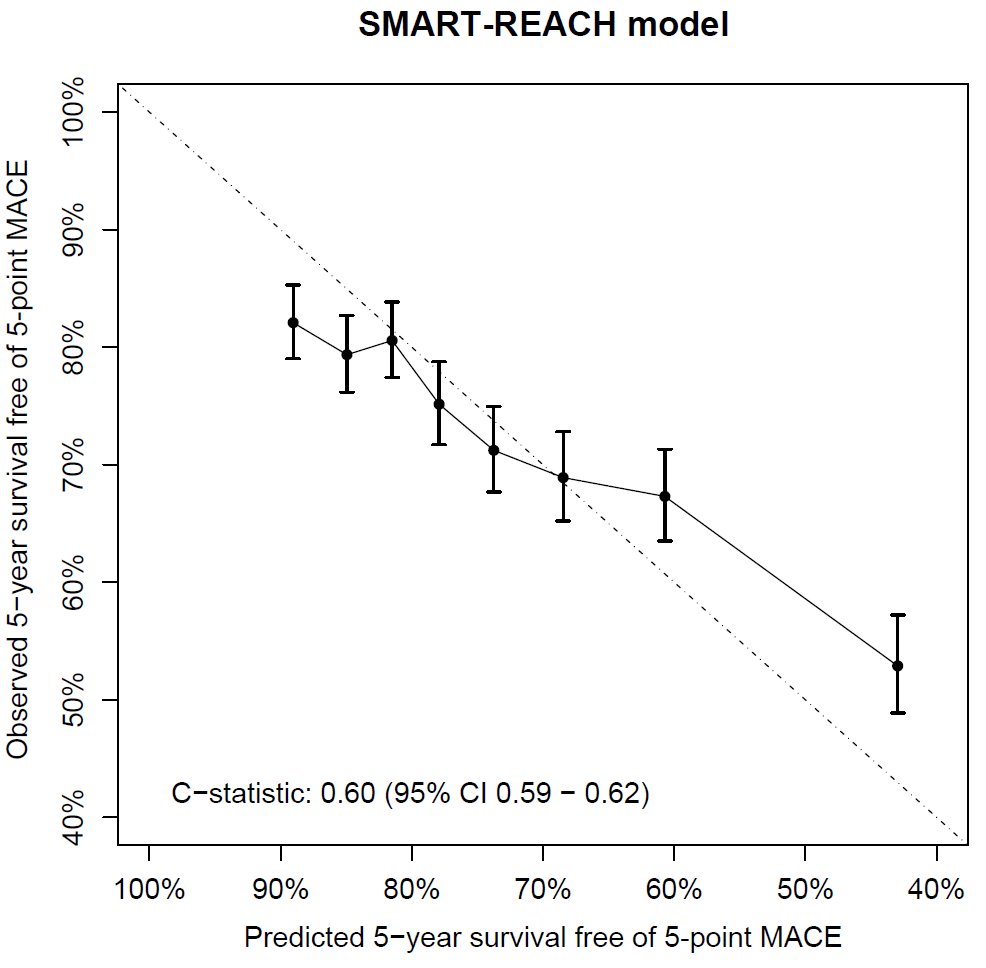


5-point MACE

Calibration in octiles of predicted risk and Harrel’s c-statistics for the SMART2 and SMART-REACH risk models. Error bars represent 95% confidence intervals. Three-point MACE is a composite of non-fatal myocardial infarction, non-fatal stroke, or cardiovascular death. Five-point MACE additionally includes coronary revascularization, and unstable angina.

Abbreviations: MACE = major adverse cardiovascular events.

**Figure S4. Lifetime benefit for 5-point MACE across CVD risk quartiles**

**
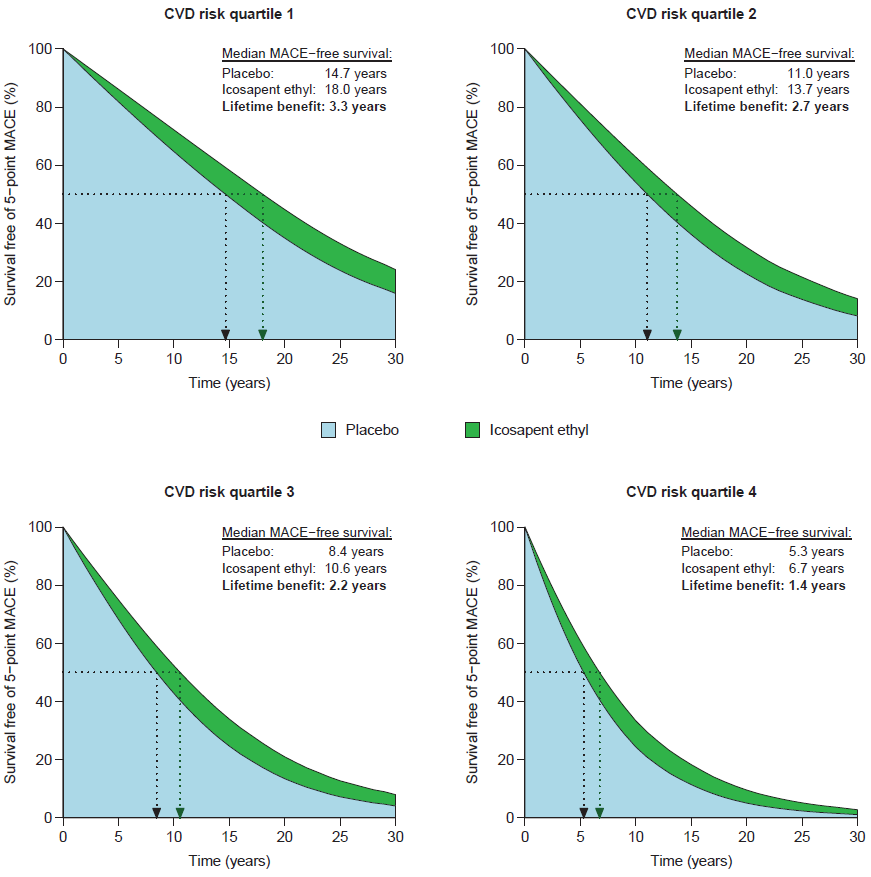
**

Average predicted survival free of 5-point MACE on placebo and on icosapent ethyl within each CVD risk quartile. Survival on icosapent ethyl was predicted by combining the recalibrated SMART-REACH model with the overall trial hazard ratio. Median MACE-free survival was defined as the time at which the survival curve crossed 50% (depicted by the dotted lines). Lifetime benefit was expressed in terms of life-years without 5-point MACE gained, and was calculated as the difference between the median MACE-free survival on placebo and icosapent ethyl respectively. Five-point MACE is a composite of non-fatal myocardial infarction, non-fatal stroke, cardiovascular death, coronary revascularization, or unstable angina.

Abbreviations: CVD = cardiovascular disease, MACE = major adverse cardiovascular events.

**Figure S5. Continuous relation between baseline risk and the effects of icosapent ethyl on 5-point MACE**

**
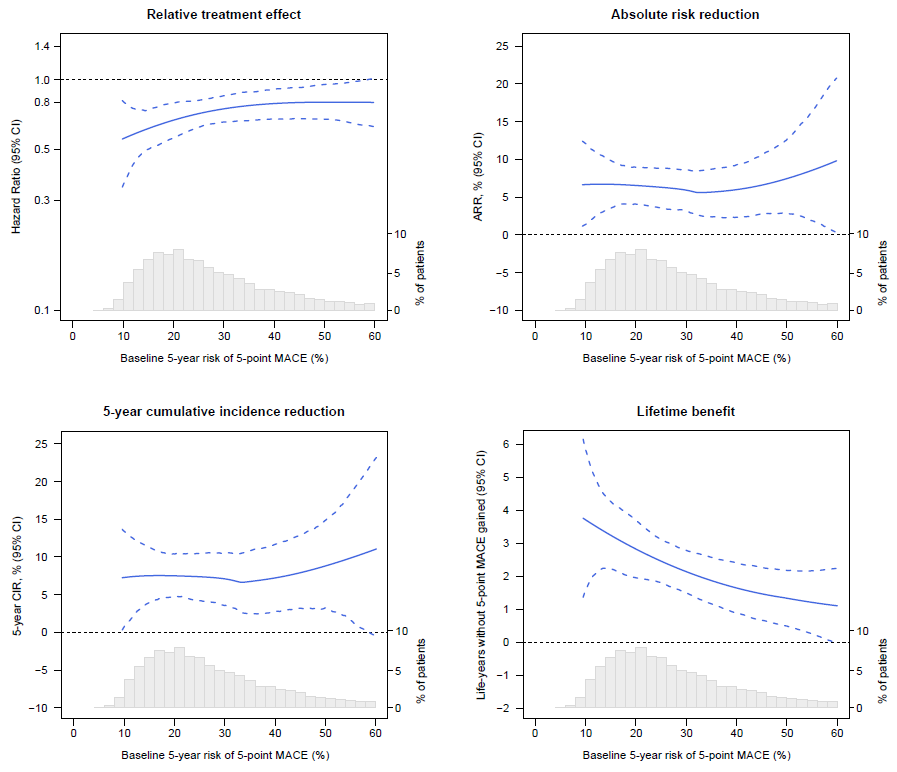
**

The continuous relation between baseline 5-year risk of 5-point MACE and the relative and absolute treatment effects of icosapent ethyl on the risk of 5-point MACE, derived from restricted cubic spline functions. The blue dotted lines denote 95% confidence intervals calculated from 10,000 bootstrap samples. The histogram (with corresponding axis at the right side of each plot) shows the distribution of baseline risk in the study population. Five-point MACE is a composite of non-fatal myocardial infarction, non-fatal stroke, cardiovascular death, coronary revascularization, or unstable angina.

Abbreviations: ARR = absolute risk reduction, CI = confidence interval, CIR = cumulative incidence reduction, MACE = major adverse cardiovascular events.

**Supplemental References**

1. Hageman SHJ, McKay AJ, Ueda P, Gunn LH, Jernberg T, Hagström E, et al. Estimation of recurrent atherosclerotic cardiovascular event risk in patients with established cardiovascular disease: the updated SMART2 algorithm. Eur Heart J. 2022;43(18):1715-1727. doi:10.1093/eurheartj/ehac056

2. Bhatt DL, Steg PG, Miller M, Brinton EA, Jacobson TA, Ketchum SB, et al. Cardiovascular Risk Reduction with Icosapent Ethyl for Hypertriglyceridemia. N Engl J Med. 2019;380(1):11-22. doi:10.1056/nejmoa1812792

3. Dorresteijn JAN, Kaasenbrood L, Cook NR, van Kruijsdijk RCM, van der Graaf Y, Visseren FLJ, et al. How to translate clinical trial results into gain in healthy life expectancy for individual patients. BMJ. 2016;352:i1548. doi:10.1136/bmj.i1548

4. Kaasenbrood L, Bhatt DL, Dorresteijn JAN, Wilson PWF, D’Agostino RB, Massaro JM, et al. Estimated life expectancy without recurrent cardiovascular events in patients with vascular disease: The SMART-REACH model. J Am Heart Assoc. 2018;7(16). doi:10.1161/JAHA.118.009217

5. Bhatt DL, Steg PG, Brinton EA, Jacobson TA, Miller M, Tardif JC, et al. Rationale and design of REDUCE-IT: Reduction of Cardiovascular Events with Icosapent Ethyl–Intervention Trial. Clin Cardiol. 2017;40(3):138-148. doi:10.1002/clc.22692

6. Hicks KA, Mahaffey KW, Mehran R, Nissen SE, Wiviott SD, Dunn B, et al. 2017 Cardiovascular and Stroke Endpoint Definitions for Clinical Trials. *Circulation*. 2018;137(9):961-972. doi:10.1161/CIRCULATIONAHA.117.033502
